# The role of motivation in talent selection in sports: insights from a comparison with personnel selection in business

**DOI:** 10.3389/fspor.2024.1463910

**Published:** 2024-12-02

**Authors:** Birte Brinkmöller, Dennis Dreiskämper, Leon Brüggemann, Oliver Höner, Bernd Strauss

**Affiliations:** ^1^ Department of Sport and Exercise Psychology, Institute of Sport and Exercise Sciences, University of Münster, Münster, Germany; ^2^ Department of Sports Psychology, Institute for Sport and Sport Science, Dortmund University, Dortmund, Germany; ^3^ Department of Sport Psychology and Research Methods, Institute of Sports Science, Eberhard Karls University of Tübingen, Tübingen, Germany

**Keywords:** achievement goal theory, assessment, athletes, personnel selection, psychological, self-determination theory, talent

## Abstract

Talent identification and selection in sports pose significant challenges, necessitating a nuanced understanding of factors influencing athletes’ elite-level potential. While physical and physiological aspects have conventionally played roles in the selection process, also other constructs of talent development have to be considered. Various talent models have included psychological aspects, especially motivation, as either moderators or catalysts. Based on empirical evidence of the relationship between motivation and performance, different views are hold in which form motivation should be used for talent selection. Considering the hierarchical model of achievement motivation and self-determination theory, the importance of different motivational dimensions in talent selection is assessed. Extending beyond sports, this study examines whether scouts, coaches, and recruiters prioritize motivational dimensions differently. Employing conjoint analysis, analytical hierarchy process, and constant-sum procedures, the research assesses weighted importance and its influences on selection decisions across domains. Results from *n* = 151 decision-makers demonstrate convergent emphasis on high intrinsic motivation and low fear of failure across domains, alongside domain-specific patterns: fear of failure and ego orientation are prioritized in sports, whereas task orientation receives greater emphasis in the business context. The assessment method (conjoint analysis, AHP and constant-sum procedure) significantly influenced outcomes, with direct ratings inflating intrinsic motivation importance (consistent with social desirability effects) while choice-based conjoint analysis revealed fear of failure as the strongest actual selection driver. The prioritization of risk avoidance (low fear of failure) over opportunity maximization (high hope for success) may suggest loss aversion bias in selection decisions. These findings inform evidence-based practice by making implicit criteria explicit and demonstrating the necessity of domain-adapted assessment approaches. Research on assessing weightings should precisely consider, which method may be most appropriate to better capture authentic decision-making.

## Introduction

Talent identification and selection remain significant challenges in sports, requiring a comprehensive understanding of various factors influencing the likelihood of athletes reaching elite levels. A successful identification of athletes is necessary to save resources, however evidence for an ideal approach is limited (1).

In addition to physical and physiological performance factors, which play a central role in talent selection, further talent development variables need to be considered. Psychological characteristics, in particular motivation, can be found in numerous talent models: while Gagné (2; Differentiated Model of Giftedness) assumes intrapersonal factors as catalysts in the process from “natural abilities” to “superior mastery of a systematically developed ability” [Hohmann (3); based on Heller (4); Munich Model of Giftedness] integrates non-cognitive personality traits as moderators of talent characteristics (predictors) and performance in the sport-specific talent model. Psychological aspects as potential talent predictors can also be found in sport-specific models (5; soccer).

Extensive literature has shown the relationship between motivation and sporting success in the last years (6–9), focusing on self-determination theory (SDT), achievement motivation and achievement goal orientation. Empirical evidence supports the relevance of motivational concepts for later success and talent development in various sports (10–12). In their systematic review on future performance in soccer, Murr et al. (13), concluded that high levels of the achievement motives *hope for success* (HS) and low levels of *fear of failure* (FF) are associated with future soccer performance. A meta-analysis (6) is limiting these relationships. Small, positive effects have been found for task orientation on future football performance (*Cohen's d* = .28). For ego orientation only a trivial effect on future football performance was shown (*Cohen's d* = .06). For self-determination, high levels of intrinsic motivation (IM) have been found to be associated with better athletic performance in tennis, mediated through psychological need satisfaction (14). Furthermore, positive and negative affect has been shown to be a mediator in the motivation-performance relationship (15). As a combination, Zuber et al. (16) found that intrinsically achievement-oriented soccer players have a higher likelihood to become professional players. Contrarily, high levels of amotivation and external regulation seem to be associated with drop-out (17, 18) or burn-out symptoms (19). Results show a relation between the motivational concepts but also the status as distinct concepts for each of them. The findings underscore the crucial role of motivation in influencing performance (10), either directly or mediated (14). However, it remains open, whether and how it should be considered in talent selection.

### Motivation theories

Recent emphasis has shifted to a combination of the hierarchical model of achievement motivation (20) and self-determination theory (SDT; 21) to examine competence from a motivational perspective (22, 23). Competence thereby describes the desire to feel effective in interactions with the environment through tasks that are appropriate to one's developmental level (24).

The three overarching concepts are the (1) achievement motivation, according to Atkinson (25) and McClelland (26), including the motive to achieve success (HS) and the motive to avoid failure (FF). These needs are seen as motivational dispositions, which explain how people perceive and evaluate situations. The second concept (2) describes motivational orientaions, which guide actions towards certain goals (22), and are most subdivided into task and ego orientation (27). Within the achievement goal theory (AGT) task orientation describes mastering a task with an individual reference norm, while ego-oriented individuals are motivated by outperforming others. The disposition of *HS* thereby leads to task goals, while *FF* is associated with an ego goal orientation. Both orientations further have an impact on the intrinsic motivation (IM; task goal orientation has a positive effect, ego goal orientation a negative one; 20). Furthermore, (3) SDT explains reasons for motivated behavior through the extend of self-determination (21) ranging from amotivation (no behavior) to extrinsic motivation (behavior based on rewards) to intrinsic motivation (behavior based on its own sake). While these aspects are not mutually exclusive, understanding their interplay is essential for effective talent identification. Within the three concepts the degree of disposition varies. While the achievement motivation is dispositional, achievement goal orientations are influenced by these and further dependent on the competence expectancy one has (20). Self-determination has the least state proportion as it is situational and a result of the evaluation of competence, autonomy, and relatedness (basic psychological needs theory; 28).

### Motivation in business

Next to the theoretical foundation of motivation in talent research as well as the empirical evidence of its relationship with performance, other domains have found a similar picture. In the business domain, the picture shows a different emphasis but similar results. Compared to other motivational concepts, IM has been widely studied in the business domain. From an organizational perspective, employees with IM might be beneficial to recruit, as IM fosters volunteering and prosocial behavior (29, 30). It further mediates the relationship between prosocial behavior and performance (31) and leads to increased engagement in organizational citizenship behavior (32, 33). Further, IM was ranked as the third important soft skill after *hardworking* and *reliable* (34). Further motivational research was investigating the relationship between need for achievement (nAch) and job performance, finding a positive relationship (35). Elliot and Harackiewicz (36) showed positive effects of learning (mastery) goal orientation and neutral or negative effects of performance goal orientation. A further distinction of performance goal orientation in performance-prove (demonstrating ability through superiority of others) and performance-avoid (trying to avoid negative outcomes) orientation revealed a positive prediction of sales performance for the first mentioned and a negative prediction of performance for the latter (37). The results show that the simultaneous adaption of different aspects may lead to optimal performance, in comparison to the complete absence of one or the other.

### Sports and business

Comparing the domains of sports and business regarding selection processes at first glance, both domains share the aim of finding the most talented people for their organization to become successful through engaging in systematic selection processes. On closer examination, additional similarities and differences emerge. Both domains use reliable methods to assess valid constructs to predict future performance, often through multi-stage processes that combine objective data through tests (e.g., 38, 39) as well as the assessment and decision of scouts, coaches and recruiters (39, 40). However, differences also arise, such as the age at which talent is typically selected and the focus on cognitive skills in business vs. physical skills in sports. Notably, parallels exist in the importance of psychological constructs (41, 42), though it remains unclear, whether these are assessed and valued similarly, potentially pointing to either convergence or divergence in selection practices. Despite these apparent parallels, citation network analyses reveal a systematic disconnection between sports and business selection research (43), limiting knowledge transfer and potentially explaining divergent approaches to assessing psychological factors such as motivation.

Although the influence of various motivational dimensions on performance has been proven in both domains, in sports as well as in business, the systematic integration of motivation into the selection process has so far been insufficient. To date, the importance of the different dimensions of motivation has only been considered unsystematically in talent selection. The correct recording and assessment of various motivational dimensions is therefore important, as it can both increase the efficiency of the selection process and ensure the long-term success and satisfaction of the selected individuals. As motives refer to internal thoughts and emotions, they are difficult to observe (e.g., 44). Although tools to assess achievement-motivated behavior by coaches exists (45), in practice coaches are still asked to assess player's psychological characteristics on unstructured evaluation sheets (46). It remains open how recruiters and scouts evaluate the importance of different dimensions of motivation in selection contexts specifically. Especially the theoretical differentiation of motivation in its different dimensions seems to be overlooked in research and practice. To date there is no evidence, which aspects of motivation are mostly prioritized by recruiters and scouts.

As in both domains the decisions of recruiters and scouts/coaches are often subjective and intuitive (47, 48), decision-makers are often not able to explicitly reconstruct their decision-process (48). Especially if different selection factors are considered, the individual importance (weighting against other factors) is often still unclear. Therefore, indirect measurements, such as conjoint-analysis (49) or multiple criteria decision-making processes (e.g., Analytical Hierarchy Process; 50) to assess the prioritization may be beneficial. Furthermore, a comparison to direct measurements can provide information about possible differences in prioritization and thus help to clarify the decision-making process for coaches, trainers and recruiters. Given these apparent parallels, a closer examination of the two domains promises first insights into shared principles and domain-specific adaptations in selection.

### Aim of the study

Based on the presented empirical evidence, factors, like different approaches to psychological aspects, such as motivation, may explain the disconnection between sports and business literature on talent selection (43). We will assess which dimensions of motivation are seen as important by decision makers to gain a deeper understanding of the priorities in selection contexts and potential differences in the prioritization of motivational dimensions. We further assess whether the importance differs between scouts/coaches and recruiters when rating the same population (job applicants or athletes) or whether the ratings are stable within decision-makers, independently of the rated population.

Because of the intuitive decision-making (48) and the difficulty to explicitly state the decision process, the importance of motivational dimensions are assessed indirectly and directly. Therefore, three different dependent variables are assessed: (a) an implicit weighting for each motivational dimension, indicating how important this dimension is relative to the other dimensions, (b) the relative importance of each motivational dimension through an indirect measurement and (c) the direct assessment of the importance of the motivational dimensions.

Therefore, our study attempts to assess (1) the perceived importance of different dimensions of motivation (i.e., hope for success, fear of failure, ego orientation, task orientation, intrinsic motivation, extrinsic motivation) of decision-makers in talent selection (Research Question 1; RQ). We further want to investigate whether the perceived importance depends on (RQ 2.1) the domains (sport vs. business), (RQ 2.2) varying expertise of the decision makers (RQ 2.3) contexts within the decision-maker or (RQ 2.4) whether they are rated directly or indirectly, via the selection of profiles. This will be investigated against teachers, which represent a baseline of decision-makers not related to sports or business.

This research aims to provide insights into the importance of different motivational dimensions in selection processes across sports and business, seeking to explore potential transferable principles that could contribute to a deeper understanding of selection strategies. This may encourage to re-evaluate existing practices, e.g., assessed motivational dimensions and assessment methods. Furthermore, the understanding of how professionals from different domains evaluate motivational dimensions may also lead to further investigation on the alignment of candidates’ qualities and the specific demands of each context as well as the goal of the selection.

## Method

### Participants

For participation in the study, three groups of individuals were recruited: coaches and scouts from the sports domain, individuals with recruitment experience from the business sector and teachers. Teachers constituted as the control group, as they are not influenced by domain-specific aspects and therefore allow for comparisons between experts and novices. Participants had to be at least 18 years old and had to be at least in the middle-to-expert-stage, i.e., a minimum of five years of experience in selection in sports or business. To be consistent, coaches and scouts had to work within team sports, e.g., football, basketball, etc. and needed to be involved in the selection of youth academies or squads, as higher selection processes are more in alignment with personnel selection in business. Recruiters needed to work in at least medium-sized companies to ensure structured selection procedures (according to 51). Teachers must have completed their teacher traineeship to make sure that they have relevant experience in their field as well. Participant collection was facilitated using the snowball sampling (52) and via social media. Snowball sampling is executed by making initial contact with personal contacts of the authors and active engagement with sports associations, businesses, and schools. Within the invitation the link for the survey was directly included as well as the invitation to distribute the survey to their own network.

### Measurement procedures

To assess the three dependent variables of the importance of the motivational dimensions different procedures were conducted: conjoint analysis, analytical hierarchy process and constant-sum procedure. All three methods assess the relative importance of the motivational dimensions, differing in the directness of the query (from indirect to direct).

#### Conjoint analysis

To assess preferences and attitudes towards profiles of players and job applicants, a choice-based conjoint analysis was conducted. Conjoint analyses, frequently utilized in marketing research to assess consumer preferences or attitudes towards products and multi-attributive concepts (49) involve examining individual responses to discern people's preferences, relative importance, or priorities regarding the features of the object through statistical techniques. In contrast to explicitly soliciting preferences, conjoint analysis conceptualizes decision-making as a process involving trade-offs among various multi-attribute products or services (53). In this study, conjoint analysis were adapted to the context of selection by evaluating profiles of both athletes and job applicants. The profiles exhibited varying manifestations across the motivational dimensions, specifically ego-orientation, task-orientation, hope for success, fear of failure, extrinsic motivation and intrinsic motivation. The gradations in motivational dimensions were demarcated as high, medium, and low, representing relative scores of questionnaires compared to “the other applicants/athletes”. Participants were tasked with choosing the most suitable profile among three options, mimicking subjective decision-making processes akin to actual selection scenarios. The full factorial design holds 729 profiles (*n* = 3^6^). The experimental design included 27 profiles, which have been calculated with R Studio. The orthogonal design shows an acceptable fit with a *d-efficiency =* .876 (54).

#### Analytical hierarchy process (AHP)

The Analytic Hierarchy Process (AHP) is a general theory of measurement (50) and is widely used for multiple criteria decision making (55). It is a method of decision making that allows for both deductive and inductive thinking without the use of syllogisms. Comparative judgments are made by comparing each element to every other element and assigning a numerical value to represent the relative importance of each element. Synthesis of priorities involves combining the judgments made at each level to arrive at an overall priority for each element in the hierarchy. It is used to derive ratio scales from both discrete and continuous paired comparisons. In the present study, elements represent the different dimensions of motivation (hope for success, fear of failure, intrinsic motivation, extrinsic motivation, ego orientation, task orientation) which are represented through items from validated questionnaires (Table 1). Every element (dimension of motivation) is given a paired comparison with every other element, resulting in n(n-1)/2 direct comparisons with *n* elements. Participants were tasked with appraising each pairwise comparison on a nine-point scale, reflecting relative importance to both sides (−4 = extremely more important [left side], 0 = identical or minimal differences in importance; 4 = extremely more important [right side]; 50, 56). A higher numerical value signifies a more substantial disjunction in significance. A 9-point-scale was used, compared to an 18-point-scale as in the original work by Saaty (50) which is common in consumer research (56). Numeric values were transformed. Comparisons were randomized. Results showed the prioritized rankings, providing a clear hierarchy of the elements based on their relative significance to recruiters, scouts and teachers. Compared to conjoint analysis, the analytical hierarchy process allows for more explicit decision making by choosing only between two individual aspects and can therefore be seen as a variable-oriented approach as every dimension is rated solely against another dimension. Preferences for motivational dimensions were, compared to conjoint analysis, assessed directly as dimensions will be rated against each other.

**Table 1:** Items for motivational dimensions and respective questionnaires for the analytical hierarchy process.

Theory	Dimension	Field	Item	Item Nr.	Questionnaire
Achievement goal theory	Task orientation	Sport	I feel most successful in sport when I enjoy learning something new.	1	Transferred from task and ego orientation at work questionnaire [TEOWQ, (120)]
		Business	I feel most successful at work when I enjoy learning something new.	5	Task and ego orientation at work questionnaire [TEOWQ, (120)]
	Ego orientation	Sport	I feel most successful in sport when I am the only one who has mastered the skill.	2	Task and ego orientation questionnaire in Sports-German [TEOSQ-D; (121)]
		Business	I feel most successful at work when I am the only one who can do the job.	1	Task and ego orientation at work questionnaire [TEOWQ, (120)]
	Hope for success	Sport	I like sporting challenges that I don't know exactly whether I can complete.	5	Achievement motive scale sport [AMS-S; (113)]
		Business	I like tasks at work that I don't know exactly whether I can complete.	5	Transferred from achievement motive scale—sport [AMS-S; (113)]
	Fear of failure	Sport	I feel uncomfortable doing something in sport if I'm not sure that I'll succeed.	21	Achievement motives scale—revised (122)
		Business	I feel uncomfortable doing a new task at work if I'm not if I am not sure that I will succeed.	21	Achievement motives scale—revised (122)
Self-determination theory	Intrinsic	Sport	I do sport because I like the feeling of being completely immersed in an activity.	25	German version of the sport motivation scale [SMS28; (123)]
		Business	I do the job because I enjoy my work.	Intrin2	Multidimensional work motivation scale (124)
	Extrinsic	Sport	I do the sport because it gives me respect from people I know.	6	German version of the sport motivation scale [SMS28; (123)]
		Business	I do this job because it gives me security.	16	Multidimensional work motivation scale (124)

To ensure the validity of the items, pre-validation was carried out. For this aim, individual items were presented to experts in the field of motivation and asked to assign the items to the different theories. Items ranked as not fully clear were rephrased through discussions with experts.

#### Constant sum procedure

To assess the explicit subjective significance assigned by recruiters, coaches and scouts to motivational dimensions, constant sum procedures was implemented as another variable-oriented approach. Participants were tasked with allocating a total of 100 points across the six dimensions of motivation depending on their subjective importance. A higher number of points represents a higher importance. The outcomes not only unveil an importance ranking for the dimensions but will also provide relative importance ratings among these dimensions.

### Sample size

The required sample size is calculated and reported for conjoint analysis. This is done for three reasons: (1) conjoint analysis will be the predominant analysis of the study, (2) the AHP has the advantage of small sample sizes to achieve statistically robust results (57) and (3) the calculated sample size for the constant sum procedure is smaller than for conjoint analysis.

Therefore, an *a priori* power analysis was conducted using cjpowR in R Studio (58). According to Hainmueller, Hopkins, and Yamamoto (59), AMCE is the most commonly examined causal quantity in conjoint experiments. An alpha of .05 and AMCE = .02 was used. Based on three levels, 27 profiles and nine tasks, results showed a total sample size of 121 respondents resulting in *n* *=* 40 respondents for each group to reach a power of .8. As the small population of scouts within higher team sports is limited, we further follow recommendations of Orme (60) to collect a representative number of the population.

### Procedure

**Figure 1:**
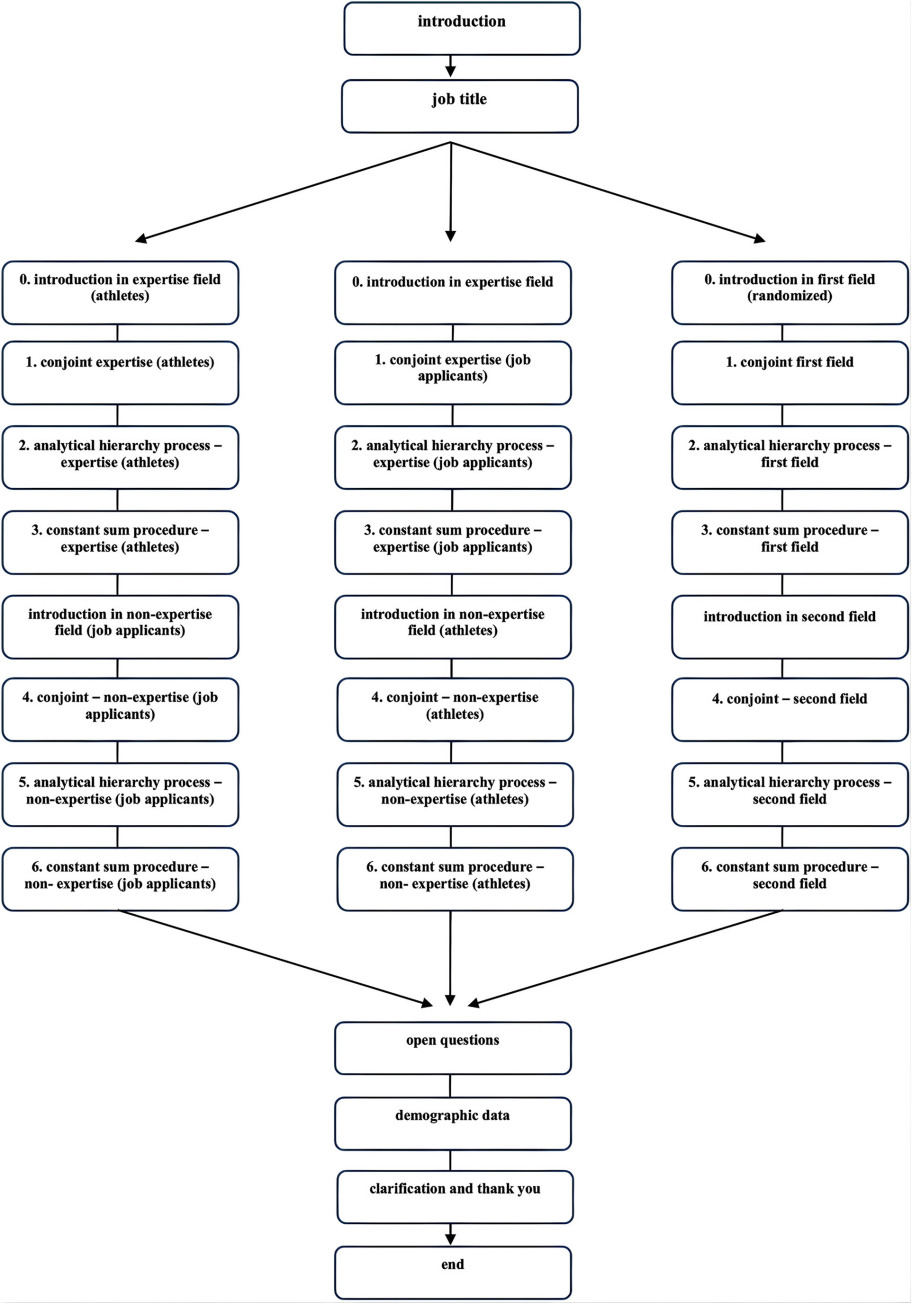
Schematic process of the survey.

The survey was administered in the form of an online questionnaire. Preliminary to the survey initiation, a comprehensive elucidation of the study's procedural aspects was provided, accompanied by a requisite privacy declaration. The questionnaire comprised several segments, which, aside from nuances in the mode of address, are analogous for recruiter, scouts, and teachers (see Figure 1). To facilitate cross-disciplinary comparisons, all three participant cohorts were presented with thematic blocks encompassing sports-related and corporate applicant scenarios. For sports scenarios, the selection for the U19 national team was chosen. The equivalent for the business context was to decide for a project-management position with three years of experience. These scenarios were chosen because both seem to be shortly before a final job position. Based on their predominant job, participants were introduced to their field of expertise. In the inaugural section, participants got an introduction to the scenario (being a recruiter/scout) and an explanation of the different dimensions of motivation. Afterwards they were presented various profiles within the conjoint analysis. Profiles were presented randomly on nine pages with three profiles each. Preferences for motivational dimensions were assessed indirectly via the selection of profiles within a decision-making process. Subsequently, the ensuing section introduced pairwise comparisons of the six motivational dimensions, employing the AHP. The final section solicits respondents’ perspectives on the relative importance of the dimensions. Subsequently, participants received an introduction to the respective other field, with again, a reminder of the explanation of the different dimensions. For teachers, the blocks were randomized, ensuring that half of the participants started with evaluating athletes and half of them with the evaluation of applicants. At the end, the collection of demographic data, encompassing variables such as gender, age, title, professional tenure, vocational training, and supplementary qualifications was ensured.

### Statistical analysis

Results were generated using R Studio. For the conjoint analysis the package *cregg* in R was used (61). Analysis of the AHP was conducted using the package *ahpsurvey* (62)*.* For AHP, consistency ratio (CR) was calculated to ensure that pairwise comparisons are consistent. A CR < 0.1 is seen as acceptable (63). If CR is >0.1, judgements were revised to reach consistency (64). Prior to conducting the inferential statistical analyses, assumption checks were performed according to the respective procedures. The significance level for all statistical tests was set at 0.05.

To answer RQ1 a-c, for the conjoint analysis, marginal means were calculated (64). These values represent the average outcome for a specific conjoint feature level, averaged across all other features. Furthermore, we estimated the average marginal component effects (AMCEs). AMCEs indicate the average change in the probability of choosing a candidate when one attribute level is switched to another (59). The relative importance and rankings for all attributes was calculated for the AHP and the constant-sum procedure for each group (scouts, recruiter, teacher) as well as their aggregation. Results are presented individually as well as grouped for all three methodological approaches.

To answer RQ 2.1 a-c (difference between domains sports vs. business) an ANOVA was calculated individually for each method (65). The dependent variable was the decision of the participants, the independent variables were the groups and motivational dimensions. If a significant result occured, the differences between the dimensions are described descriptively post-hoc.

To answer RQ 2.2 a-c, Chi-square tests were calculated for “applicants” and “athletes” for each method.

To answer RQ 2.3 a-c, Chi-square tests were calculated for “scouts” and “recruiter” for each method.

To answer RQ 2d, a ranking-order for each participant for each method (*n* = 3) and for each rating group (applicants, athletes; *n* = 2) was calculated. Chi-square tests were calculated by the differences between each method within each rating group (3 × 2).

## Results

The results are presented for each research question individually and divided between the three methods which were used, respectively. Prior to conducting chi-square tests, we examined whether no more than 20% of cells from the expected frequencies table yielded values <5, as this violates a key assumption of the chi-square test (66, 67). In cases where this condition was not met, we applied a Monte Carlo simulation procedure (68) with 100,000 replications to estimate the exact *p*-value. The method used is stated in the presentation of the corresponding results: chi-square tests without degrees of freedom were corrected with a Monte Carlo simulation. When degrees of freedom are present, assumptions were met for chi-square tests. *Cramer's V* (*V*) was calculated as an effect size and can be interpreted according to Cohen (69). For the AHP, consistency ratio was checked. All elements, which did not reach the threshold of a consistency ratio > 1 were removed from the dataset. The analyses show that the teacher's evaluations were less consistent than those of the scouts^1^ and recruiters. This indicates the presence of an expertise effect in the two focal groups and confirms the suitability of the teachers as a control group. Detailed results concerning the teacher group, along with additional analyses, are provided in the Supplementary Materials. Accordingly, the following section reports and discusses only the results pertaining to the research questions including scouts and recruiters. All data and analysis code are publicly available on the Open Science Framework (https://uni.ms/fajlo).

### Participants

Data collection occurred between December 2024 and July 2025. Participants were recruited via snowball sampling (52) and social media advertisement (e.g., LinkedIn). Initial contact was established through the authors’ professional networks. To incentive participation, respondents could opt to enter a prize draw for shopping vouchers. Participation was voluntary, and respondents could withdraw at any time without penalty. The survey included mandatory questions to decrease the amount of missing data (for a critical discussion, see 70). A total of *N* *=* 403 participants filled out the online-survey, out of which *n* *=* 151 datasets were included in the analysis. Participants were excluded from the analysis if they have not met the inclusion criteria (team sports for scouts and medium-sized companies for recruiters), outlined in the method section, or did not finish the survey. The number of recruiters and teachers is in line with the calculated sample size (*n* = 40). The number of scouts is reduced, however due to the smaller population of this group, the number represents the population. The demographic characteristics of the participants are presented in Table 2.

**Table 2:** Demographic characteristics of participants divided by groups.

Characteristics	Scouts	Recruiters	Teachers	Total
*n*	27	50	74	151
Age (*M* *±* *SD*, years)^a^	42.86 (13.49)	51.66 (9.76)	48.93 (13.44)	48.67 (12.66)
Experience (*M* *±* *SD*, years)	16.45 (9.61)	15.94 (8.49)	17.35 (11.01)	16.72 (9.95)
Gender (% female)^a^	24.14	62.00	79.73	63.40

^a^ Significant group differences between scouts and recruiters. Age: ANOVA [*F*(2,72.178) = 4.747, *p* = .012]; Games-Howell *post-hoc* [−8.8, 95%-CI (−15.7−1.87)]; Gender: *χ^2^*(2) = 28.986, *p* < .001.

For the subsample of scouts, most of them worked in soccer (*n* *=* 9), followed by basketball (*n* *=* 4) and volleyball (*n* *=* 3, Supplementary Table S1). All participants not working in team sports were excluded from the study (*n* *=* 42). For the recruiters, 34% worked in medium-sized (50–249 employees) companies (66% in big companies, i.e., >250 employees). Recruiters from small companies [<49 employees; (51); *n* *=* 14] were excluded due to the mostly unstructured selection procedures in these companies.

### Importance of motivation in selection contexts (RQ 1)

#### Conjoint analysis

**Figure 2:**
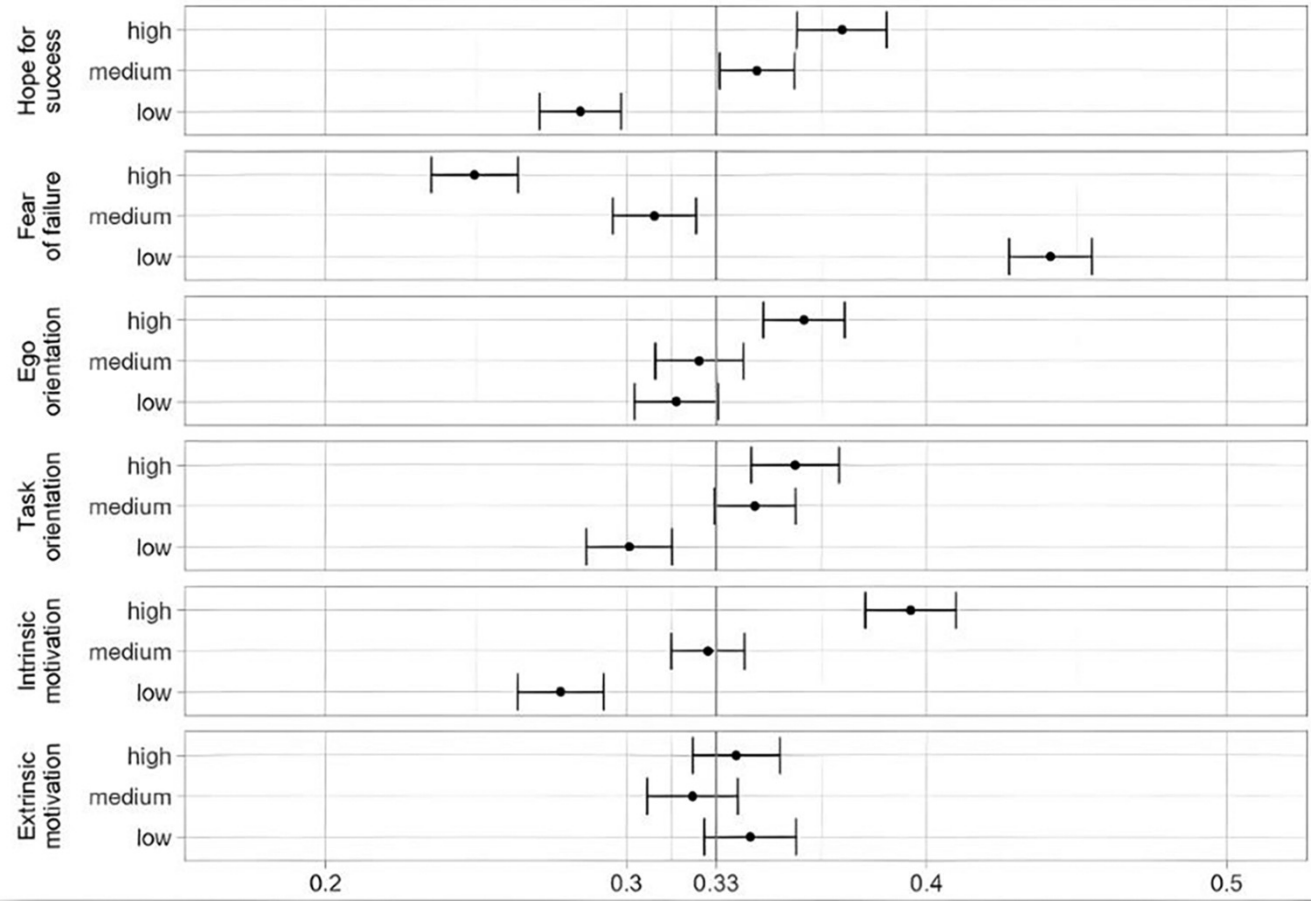
Marginal means for the overall sample.

The conjoint analysis (CA) showed differentiated preference patterns across the motivational dimensions. Marginal means (MM) were calculated and represent the average probability of profile selection for specific attribute levels averaged across all other features. The mean MM for three levels is .33. A *MM* = .45 means that, on average, profiles containing this attribute level are chosen 45% of the time, which is above the baseline of *MM* = .33 and therefore preferred. Results indicated significant differences between levels for most dimensions, except extrinsic motivation (Figure 2). Significantly distinct preferences between all levels (high vs. medium vs. low) emerged for hope for success, fear of failure and intrinsic motivation. For hope for success, low levels were preferably rejected (*MM* = .285, *p* < .001) compared to a preference for medium (*MM* = .344, *p* < .001) and high levels (*MM* = .372, *p* < .001). Regarding fear of failure results show a preference for low fear of failure (*MM* = .441, *p* < .001), whereas high levels of this dimension were rejected (*MM* = .249 *p* < .001). Regarding intrinsic motivation, an inverse pattern emerged: results showed a rejection of low levels (*MM* = .278, *p* < .001) while high intrinsic motivation was preferred (*MM* = .395, *p* < .001). For ego orientation, high levels were preferred (*MM* = .359, *p* < .001) compared to medium and low levels. For task orientation, low levels were rejected (*MM* = .301, *p* < .001) compared to medium or high levels.

**Figure 3:**
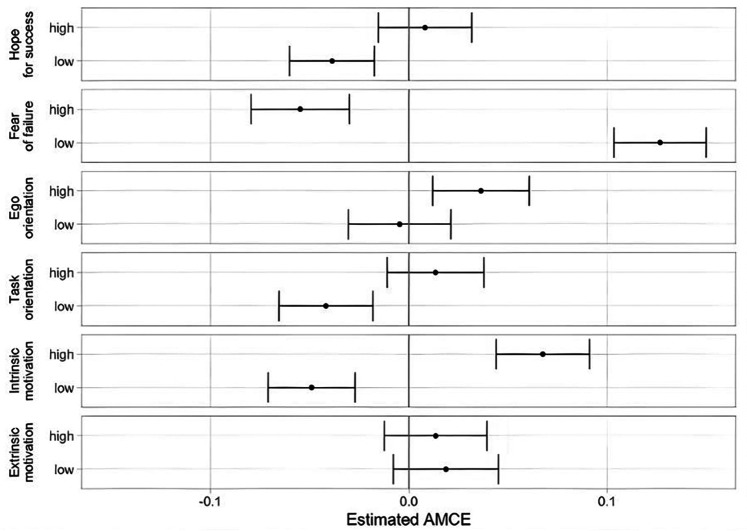
Estimated AMCE for the overall sample. AMCEs are calculated with the medium level as a reference.

The Estimated Average Marginal Component Effects (AMCEs) indicate the average change in the probability of choosing a candidate when switching between attribute levels, independent of the other attributes. An AMCE of .04 therefore means, that, on average, the presence of this attribute level increases the probability of a respondent choosing the candidate by 4% compared to the baseline (medium level). Results are presented in Figure 3. Using medium levels as the reference category (*AMCE* = 0.00), significant effects were observed for low levels of hope for success (*AMCE* = −.039, *p* < .001), high (*AMCE* = −.055, *p* < .001), and low levels of fear of failure (*AMCE* = .127, *p* < .001), high levels of ego orientation (*AMCE* = .036, *p* < .001), as well as low task orientation (*AMCE* = .036, *p* < .001). Additionally, distinct effects emerged for high intrinsic motivation (*AMCE* = .068, *p* < .001), and low intrinsic motivation (*AMCE* = −.049; *p* < .001).

#### Conjoint analysis, analytical hierarchy process & constant-sum procedure

In addition to the MM and AMCE, rankings and the relative importance of dimensions were calculated for all three methods. For CA, the relative importance by attribute (attribute partworths), represents the score, how much every dimension influences the decision-makers selection. For AHP and constant-sum procedure (CSP), percentages show the relative importance respondents assign to each attribute compared to the others. While the results of CA and AHP reveal a similar prioritization pattern, the CSP yielded yet another distinct rating pattern. Results of the CA, AHP and CSP are presented in Table 3. The average preferences per level, as well as subgroup ranking orders of all methods can be found in the Supplementary Material (Figure S1–S10).

**Table 3:** Relative importance and ranking of dimensions divided by method.

Motivational dimension	CA	AHP	CSP
Fear of failure	39.45% (1)	19.56% (2)	19.40% (2)
Intrinsic motivation	25.36% (2)	24.55% (1)	17.48% (4)
Task orientation	12.01% (3)	17.46% (3)	9.64% (5)
Hope for success	10.19% (4)	16.26% (4)	19.39% (3)
Ego orientation	8.92% (5)	13.90% (5)	27.78% (1)
Extrinsic motivation	4.08% (6)	8.27% (6)	6.31% (6)

Ranking is based on the results of the CA (decreasing) for a better comparison. Number in brackets represent the ranking within each method.

### Difference between sports and business domain (RQ 2.1)

#### Conjoint analysis

**Figure 4:**
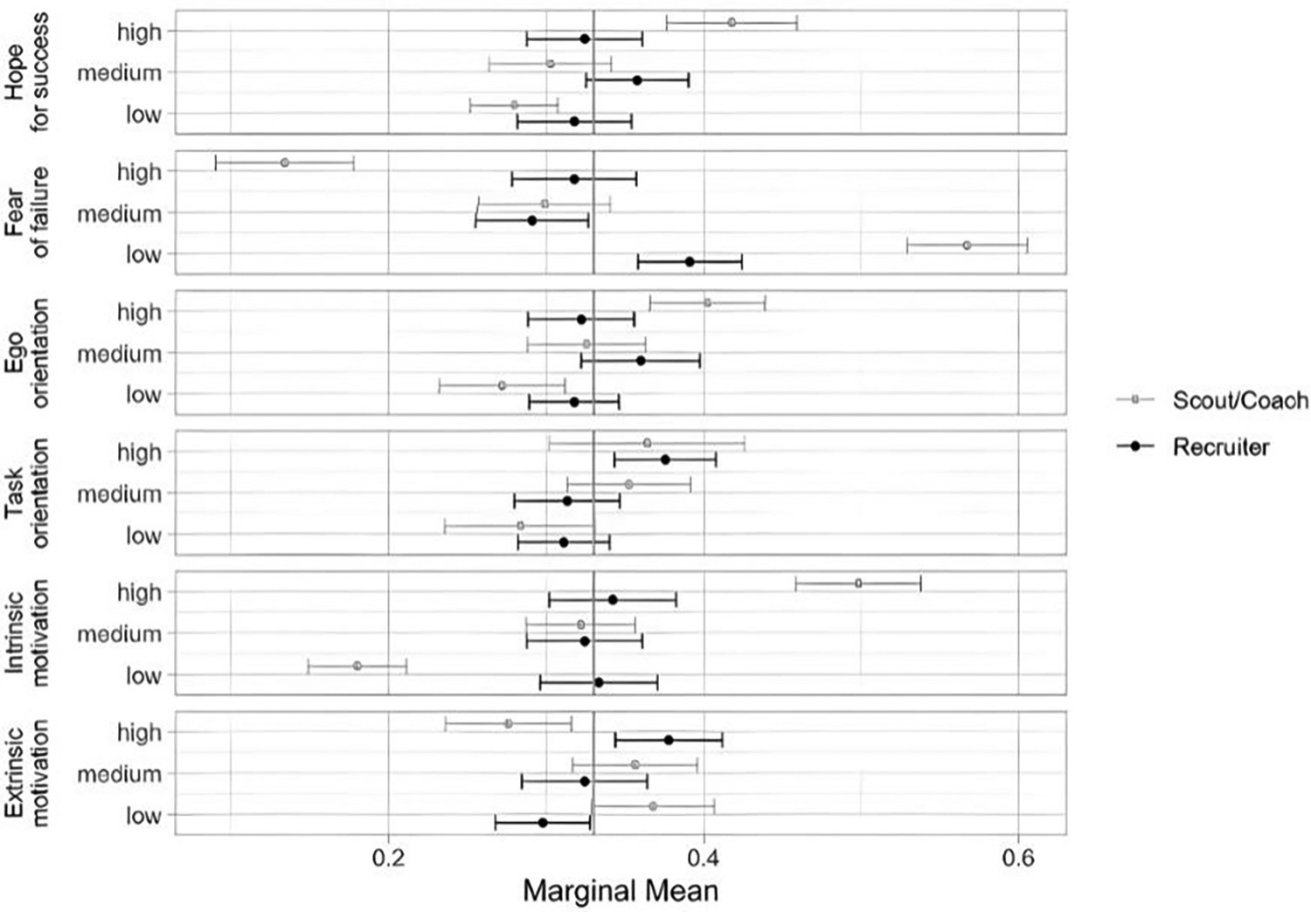
Subgroup marginal means.

The mixed factorial ANOVA revealed that adding the interactions with the variable *group* significantly improved the model (*F*(13, 2,107) = 9.32, *p* < .001, partial *η*^2^ = .054), indicating that the relative importance of motivational dimensions varied depending on whether scouts were evaluating athletes or recruiters were evaluating applicants. Figure 4 shows the Subgroup Marginal Means. The Subgroup AMCEs as well as the Average Preferences for Level can be found in the Supplementary Material (Supplementary Material Figure S11–S13). While most of the attribute levels were considered equally important, some show significant differences in their importance:

Scouts demonstrated a stronger preference for high levels of hope for success, low levels of fear of failure, high levels of ego orientation and high levels of intrinsic motivation compared to recruiters. In contrast, recruiters rated high extrinsic motivation more favorably than scouts. Furthermore, scouts rejected high levels of fear of failure as well as low levels of intrinsic motivation significantly stronger than recruiters. Overall, there is a greater differentiation between the levels of dimensions for scouts compared to recruiters.

#### Analytical hierarchy process

**Figure 5:**
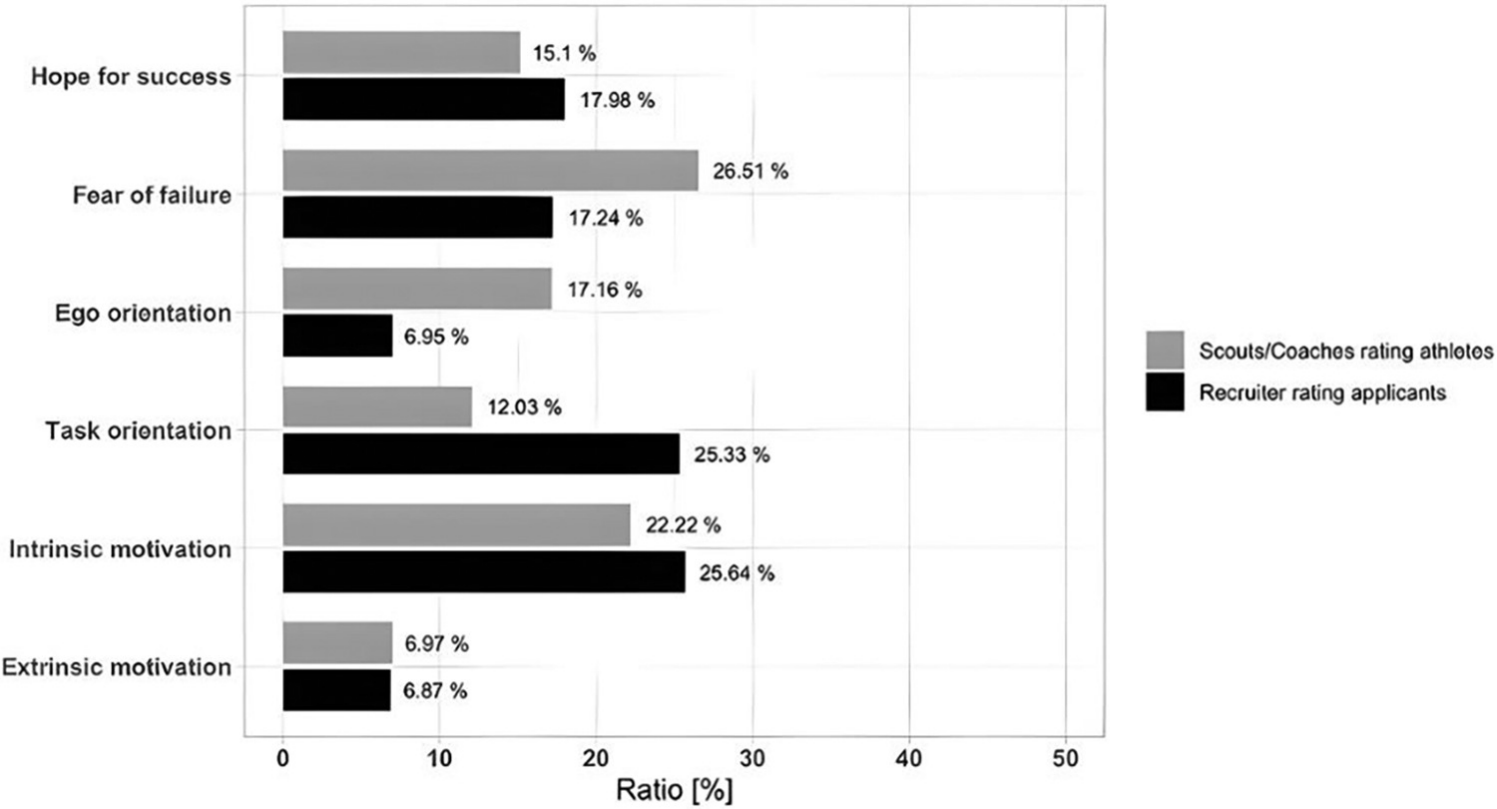
Comparison of relative importance of dimensions (AHP, in %) between sports and business contexts.

The mixed factorial ANOVA revealed a significant interaction effect between group and motivational dimension (*F*(5, 438) = 20.23, *p* < .001, partial *η*^2^ = .19), demonstrating that the relative importance of motivational dimensions varied depending on whether scouts rated athletes or recruiters rated applicants. Specific differences between groups emerged for fear of failure (*p* < .001, *η*^2^ = .216), ego (*p* < .001, *η*^2^ = .373), and task orientation (*p* < .001, *η*^2^ = .313). The average scores per dimension for both domains are presented in Figure 5.

#### Constant-sum procedure

**Figure 6:**
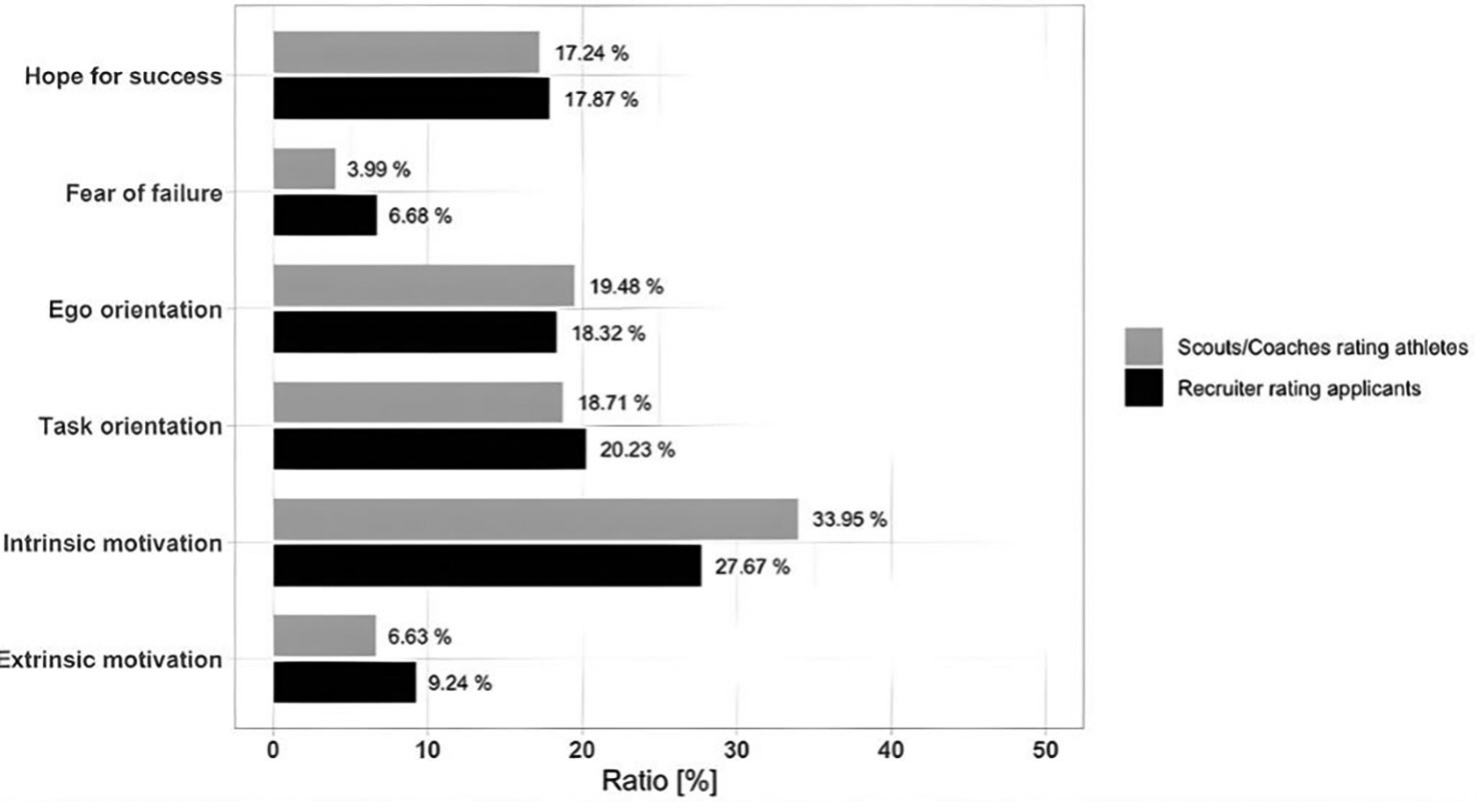
Comparison of relative importance of dimensions (CSP, in %) between sports and business contexts.

The CSP also revealed significant group differences. The ANOVA showed a significant group × dimension interaction (*F*(5,462) = 2.833, *p* = .016, partial *η*^2^ = .03). The interaction effect suggests that scouts and recruiters differed in their prioritization patterns. For both groups, intrinsic motivation seems to be the most important dimension (Figure 6). Specific group differences were identified for fear of failure (*p* = .015, *η*^2^ = .075) and intrinsic motivation (*p* = .022, *η*^2^ = .066). While fear of failure seems to be more important for recruiters, intrinsic motivation seems to be more important for scouts.

### Differences between decision-makers (scouts evaluating athletes vs. recruiter evaluating athletes & scouts evaluating applicants vs. recruiter evaluating applicants) (RQ 2.2)^2^

#### Conjoint analysis

***Athletes*** Chi-square tests revealed mostly non-significant differences between scouts and recruiters when both evaluated athletes. Significant differences only emerged for fear of failure (*χ*^2^ = 19.19, *p* < .01, *Cramer's V* = .49) and extrinsic motivation (*χ*^2^ = 12.76, *p* = .023, *V* = .40). Fear of failure was higher ranked by scouts, while extrinsic motivation was higher ranked by recruiters.

***Applicants*** In contrast, when both recruiters and scouts evaluated applicants, no significant differences were observed between the two groups (all *p* > .05).

#### Analytical hierarchy process

***Athletes*** When evaluating athletes, a significant difference between scouts and recruiters was only found for task orientation (*χ*^2^ = 13.99, *p* = .011, *V* = .43), with recruiters rating this dimension as more important than scouts.

***Applicants*** When both groups evaluated job applicants, a significant difference was found for extrinsic motivation (*χ^2^* = 10.74, *p* = .030, *V* = .38), with scouts placing more emphasis on this dimension.

#### Constant-sum procedure

***Athletes*** Substantial differences between scouts and recruiters emerged when rating athletes across multiple dimensions. Significant differences were found for hope for success (*χ*^2^ = 15.10, *p* < .01, *V* = .44), ego orientation (*χ*^2^ = 18.89, *p* < .001, *V* = .49), task orientation (*χ*^2^ = 15.77, *p* < .01, *V* = .45), and intrinsic motivation (*χ*^2^ = 18.56, *p* < .001, *V* = .48), with medium to large effect sizes. The pattern of differences showed that scouts rated ego orientation and hope for success as more important than recruiters, while recruiters placed greater emphasis on fear of failure and intrinsic motivation compared to scouts.

***Applicants*** When evaluating job applicants, no significant differences between the groups were observed (all *p* > .05).

### Differences between target groups (scouts evaluating athletes vs. scouts evaluating applicants & recruiters evaluating athletes vs. recruiters evaluating applicants) (RQ 2.3)^**3**^

#### Conjoint analysis

***Scouts*** Chi-square tests revealed significant within-group differences only for the dimension fear of failure (*χ^2^* = 15.59, *p* < .01, *V* = .52). Scouts rated this dimension as more important when evaluating athletes than when evaluating job applicants.

***Recruiters*** Similarly, recruiters only showed a significant difference in their rating of fear of failure (*χ^2^*(5) = 11.3, *p* = .046, *V* = .34), with this dimension being rated as more important when evaluating athletes compared to job applicants.

#### Analytical hierarchy process

***Scouts*** Significant differences emerged for fear of failure (*χ^2^* = 16.93, *p* < .01, *V* = .56), ego orientation (*χ^2^* = 29.91, *p* < .001, *V* = .74), task orientation (*χ^2^* = 30.38, *p* < .001, *V* = .75), and intrinsic motivation (*χ^2^* = 12.09, *p* = .019, *V* = .47). Scouts rated fear of failure and ego orientation as more important when evaluating athletes, while task orientation and intrinsic motivation were considered more important when evaluating job applicants.

***Recruiters*** Recruiters showed similar within-group differences for fear of failure (*χ^2^*(5) = 15.78, *p* < .01, *V* = .4), ego orientation (*χ^2^*(5) = 46.58, *p* < .001, *V* = .69), task orientation (*χ^2^*(5) = 43.49, *p* < .001, *V* = .67), and extrinsic motivation (*χ^2^* = 11.93, *p* = .023, *V* = .35). Recruiters placed greater emphasis on fear of failure, ego orientation, and extrinsic motivation when evaluating athletes, whereas task orientation was rated as more important when evaluating job applicants.

#### Constant-sum procedure

***Scouts*** Scouts showed no significant differences in their ratings between athletes and job applicants (all *p* > .118).

***Recruiters*** Recruiters demonstrated significant within-group differences for ego orientation (*χ^2^* = 16.71, *p* < .01, *V* = .41), task orientation (*χ^2^*(5) = 21.12, *p* < .001, *V* = .46), and intrinsic motivation (*χ^2^* = 12.83, *p* < .01, *V* = .36). Recruiters rated ego orientation as more important when evaluating athletes, while task orientation and intrinsic motivation was considered more important when evaluating applicants.

### Differences between assessment methods (RQ 2.4)^**4**^

To examine differences in ranking outcomes across assessment methods, average rankings were computed by assigning point values corresponding to each rank position, with totals subsequently divided by the number of participants (Table 4). Lower numerical values indicate higher rankings; for example, a value of 3.9 for hope for success in the athlete evaluation condition indicates that participants assigned this dimension an average rank of 3.9 out of six ranks. Chi-square tests were conducted to evaluate differences between the three assessment methods (CA, AHP, CSP) and between the two target groups (athletes and applicants). The analysis revealed statistically significant differences between assessment methods for both target populations across all motivational dimensions examined (Table 5).

**Table 4:** Average rankings per dimension separated by assessment method.

Motivational dimension	Group	CA	AHP	CSP
Hope for success	Athletes	3.90	3.59	2.48
	Applicants	3.15	3.12	2.78
Fear of failure	Athletes	2.64	2.51	4.95
	Applicants	3.37	3.27	4.92
Ego orientation	Athletes	3.39	2.56	2.04
	Applicants	3.52	5.22	2.54
Task orientation	Athletes	3.44	4.37	2.95
	Applicants	3.50	2.29	2.01
Intrinsic motivation	Athletes	3.20	2.36	1.67
	Applicants	3.48	1.95	1.53
Extrinsic motivation	Athletes	3.54	5.05	4.06
	Applicants	3.16	4.92	4.26

**Table 5:** *χ*^2^ statistics for method comparison.

Motivational dimension	Group	*χ^2^*	*df*	*p*	*Cramer's V*
Hope for success	Athletes	84.64	10	<.001	.31
	Applicants	53.14	10	<.001	.25
Fear of failure	Athletes	173.60	10	<.001	.45
	Applicants	124.58	10	<.001	.38
Ego orientation	Athletes	53.46	10	<.001	.25
	Applicants	178.07	10	<.001	.45
Task orientation	Athletes	74.11	10	<.001	.29
	Applicants	87.57	10	<.001	.32
Intrinsic motivation	Athletes	90.66	10	<.001	.32
	Applicants	145.44	NA^a^	<.001	.41
Extrinsic motivation	Athletes	111.94	10	<.001	.36
	Applicants	142.35	10	<.001	.40

^a^ Monte-Carlo simulation with 100,000 repetitions was executed.

## Discussion

This study investigated the relative importance of various motivational dimensions, i.e., hope for success, fear of failure, ego orientation, task orientation, intrinsic motivation, and extrinsic motivation in selection contexts rated by experts in selection and scouting. Specifically, we examined the overall importance of these dimensions within selection contexts (RQ1), whether the perceived importance of these dimensions differs across contexts (sports vs. business; RQ 2.1), between decision-maker groups (scouts vs. recruiters; RQ 2.2), and depending on the target group (athletes vs. applicants; RQ 2.3). From a methodological perspective, we also analysed whether the assessment method (CA, AHP and CSP) influences the evaluation of these dimensions (RQ 2.4). Overall, the results indicate that high levels of intrinsic motivation and low levels of fear of failure are generally viewed as favourable across participants. This pattern is particularly pronounced in the sports context, with scouts placing greater emphasis on fear of failure when evaluating athletes compared to recruiters evaluating applicants. However, the importance of dimensions seems to be partly dependent of the assessment method, as results regarding the priority differ between all three methods.

A central contribution of the present study lies in its decision-oriented perspective: rather than examining motivational dimensions as performance predictors (for an overview, see 71), the focus was on how decision-makers weight motivational information when making selection judgements. Selection decisions often reflect subjective heuristics and professional norms rather than empirically established relationships between motivational constructs and performance (72–74), making these weighting patterns crucial for understanding real-world selection processes. By contrasting sports and business, domains sharing high-stakes selection under uncertainty but differing in performance evaluation logics and temporal dimensions, the present study allows for a differentiation between domain-general and domain-specific selection heuristics (75). By isolating the relative importance of individual dimensions, practitioners can identify which motivational aspects drive their decisions, audit these weightings against empirical performance relationships, and recognize potential biases such as loss aversion. By comparing these weightings, the study reveals which motivational patterns reflect general expert judgement principles vs. contextual performance demands, leading to methodological implications. Domain-general weightings (consistent across contexts) likely reflect fundamental human judgment heuristics or robust empirical relationships, whereas domain-specific patterns indicate that valid selection criteria must be calibrated to contextual performance demands, suggesting that universal assessment protocols may be insufficient for specialized talent domains.

### Fear of failure and hope for success

Results show an overall emphasis on achievement motivation, especially fear of failure, which was emphasized preferably in the sports context. Scouts reject high levels of fear of failure in their domain, compared to recruiters in the business context (RQ2.1). Furthermore, scouts rated fear of failure as more important than recruiters when rating athletes (RQ 2.2) and both, scouts and recruiters, rated this dimension higher in athletes compared to applicants (RQ 2.3). Regarding hope for success, only the conjoint analysis showed a preference for high levels in sports (RQ 2.1) and a preference within scouts when rating athletes (RQ 2.2).

Structural differences between domains may explain fear of failure's prominence in sports. The consequences of underperformance differ significantly between contexts: in sports, athletes are subject to constant selection and de-selection processes based on single performance instances, whereas in business, performance feedback tends to be cumulative and less abrupt in its consequences (76). This contrast reflects a fundamental difference between short-term vs. long-term performance evaluation between both contexts. In addition, sports activities usually take place in public, meaning that performances can be continuously evaluated by other people. At the same time, it has been shown that athletes in high-performance areas have a strong “athlete identity”, meaning that they strongly identify with their role as athletes and the associated values and social networks (77). Although other groups may also identify with their job-role, such as an “academic identity” (78), immediate evaluation and consequences are less present. Taken together, these two aspects could lead to a greater relevance being attached to the fear of failure, as failure may have a stronger effect on a person due to their athletic identity in comparison to the business context.

Fear of failure has been shown to have particularly detrimental effects in sports (79). It is associated with avoidance behaviour and performance-avoidance goals (80–82), maladaptive self-talk (83), and self-handicapping strategies such as reduced effort or excuse-making (84, 85). Behavioural outcomes of these patterns, such as avoidance behaviour or negative self-talk are often observable to coaches and evaluators, which may explain why scouts weight this dimension more heavily. Moreover, fear of failure has been shown to possess predictive value for future performance outcomes in sports (86, 87), further highlighting its practical relevance. Within the business context, fear of failure predominantly plays a role in the context of entrepreneurship (e.g., 84) where success and failure are easier to measure and more visible compared to individual job performance in companies (89).

Although high and even medium levels of hope for success have been shown to be beneficial for sports performance (86) and work performance (90) rather less emphasis was placed on this dimension. The prioritization of low fear of failure over high hope for success in selection decisions can be explained through loss aversion. According to Prospect Theory, losses are perceived as psychologically more impactful as equivalent gains (91, 92). Applied to recruitment, scouts and recruiters may perceive the potential negative consequences of selecting someone with high fear of failure as more threatening than the missed benefits of not selecting someone with high levels of hope for success. Since personnel decision-makers are subject to the same cognitive biases as other individuals (72), they systematically prioritize risk avoidance over opportunity maximization. This pattern was particularly evident in the divergence between assessment methods: while direct constant-sum ratings emphasized intrinsic motivation, potentially reflecting social desirability given the dimensions’ positive connotation, indirect choice-based conjoint analysis revealed fear of failure as the stronger actual selection driver, particularly in sports contexts. This discrepancy suggests that selection decisions are guided by implicit heuristics that remain largely untouched in explicit self-reports.

### Ego and task orientation

Within the achievement orientation, task and ego orientation have been shown to play a medium important role in selection contexts (RQ 1): task orientation appeared to be particularly important in the business context while high levels of ego orientation were found to be more relevant in sports (RQ 2.1). When comparing scouts and recruiters, scouts placed more emphasis on ego orientation in the evaluation of athletes (RQ 2.2). Comparing the evaluation of athletes and applicants, both scouts and recruiters emphasized ego orientation for athletes, while task orientation was preferred for applicants (RQ 2.3).

This may be due to the different structural and motivational demands across contexts. In sports, success is measured directly through competition, where winning or losing against an opponent is the most salient outcome. In business settings, however, individuals are typically not evaluated through direct competition but rather through the quality and effectiveness of their task completion (76). Here, success is often defined by achieving long-term objectives, solving complex problems, or contributing to broader organizational goals. As such, task orientation may be more functional in these learning- and development-oriented environments (93). This is in line with research showing a positive relationship between learning goal orientation and job performance (37).

Furthermore, while there are still competitive elements in the workplace, such as promotions or internal advancement opportunities, these are usually based on the evaluation of how well a task is completed rather than on a head-to-head comparison (94). This distinction may explain why recruiters place greater value on task orientation, especially when evaluating applicants, and why task orientation emerged as a central dimension in the business context overall. Ultimately, this may reflect a differentiation between short-term, outcome-based judgments in sports and long-term, process-focused evaluations in economic contexts.

Additionally, the medium emphasis on task orientation in sports is in line with empirical evidence: inconsistent findings regarding the relationship between task orientation and success exist. While Kavussanu et al. (95) found associations of task orientation with current performance and Höner and Feichtinger (86) with future performance, several studies (87, 96, 97) did not find significant relationships with future performance.

For ego orientation no relationship was found with current nor with future performance in soccer (86), whereas Coelho e Silva et al. (98), found higher levels of ego orientation in higher performing and Wachsmuth et al. (87), in older athletes. Considering that scouts are those selecting athletes in higher teams, their view on the importance of ego orientation may lead to such differences between higher and lower performing athletes, though whether this emphasis is empirically justified or tradition-based remains an open question for validation research.

### Intrinsic and extrinsic motivation

Across all conditions in the present study, intrinsic motivation was consistently rated as highly important, underscoring its perceived relevance in both sports and business selection contexts. Extrinsic motivation was consistently rated as less important (RQ 1). Especially in the business context, intrinsic motivation seems to play an important role: recruiters rated intrinsic motivation as more important for athletes than scouts (RQ 2.2). Additionally, this dimension was rated more important in applicants, both from scouts’ and recruiters’ perspective (RQ 2.3).

These findings align with a substantial body of literature (e.g., 16, 29, 30) emphasizing the central role of intrinsic motivation in driving effective performance across contexts. Based on SDT (21), intrinsic motivation fuels the direction, intensity and persistence of motivated behaviour (99). When people find a task enjoyable, they choose to participate in the task, representing an aspect of intrinsic and identified motivation that enhances performance (100). This may therefore lead to increased practice, for example**.** Second, intrinsically motivated people may invest more effort in tasks, such as greater physical investment in sports or investing more cognitive resources in cognitively demanding tasks in the business context. Furthermore, enjoyable tasks lead to a greater persistence, resulting in better job performance (31) and potentially consistent practice in sports. Research has repeatedly shown that individuals with higher levels of intrinsic motivation tend to demonstrate greater effort, and productivity, all of which are predictive of improved job performance (101). In organizational settings, intrinsic and identified forms of motivation have been linked not only to increased engagement but also to enhanced creativity and job performance (102). Given these consistent findings across the literature, as well as the popularity of the concepts “intrinsic motivation” throughout the population, it further confirms that both recruiters and scouts regard intrinsic motivation as a core attribute when evaluating candidates or athletes. These results reinforce the theoretical and practical significance of intrinsic motivation as a universal criterion in selection-related decision-making. The low importance placed on extrinsic motivation may be explained by the nature of jobs (both in sports and business) where earning money is necessary. Therefore, extrinsic motivation is somehow always present when applying for a job and may therefore have been considered as a less important attribute for successful selection decisions.

### Differences between scouts and recruiters

Beyond individual decision-making, the comparison between recruiters and scouts also provides insight into domain-specific selection cultures. Differences in motivational weightings likely reflect shared professional norms regarding what constitutes “talent” or “potential” within a given field. Differences between scouts and recruiters emerged primarily in their evaluations of athletes, while their assessments of applicants were either highly similar or showed no consistent pattern. This asymmetry may reflect the differing backgrounds and context-specific expertise of the two groups. Scouts and coaches are often not employed full-time in talent identification roles (103), and many have experience outside the sports sector, including possibly familiarity with business environments. This may allow them to form more grounded judgments about applicants in a non-sports context, although both groups simultaneously fulfil the role of employees in organizations. In contrast, recruiters typically possess extensive expertise in organizational settings but may lack the domain-specific knowledge required to accurately assess athletic potential. They may be unfamiliar with (elite) sport-specific performance indicators or the predictive cues for long-term athletic development. These may even reflect the focus of both groups: while the developmental aspect may play a greater role for scouts, recruiters are mostly looking for current job performance. Furthermore, sports cultures emphasizing competitive success naturally prioritize ego orientation and fear of failure, while business cultures valuing sustained development prioritize task orientation. Cross-domain comparison thus serves as a mirror that makes these mainly implicit norms visible (72).

### Method-specific outcome patterns

The current study also highlights the importance of the assessment method used to evaluate the importance of motivational dimensions. While intrinsic motivation was ranked the highest in the CSP, fear of failure was ranked the highest, at least for athletes, when assessed through CA. For AHP, results are between the results of the other two methods. This is in line with previous work from other disciplines, comparing direct and indirect measures for preferences (104). Social desirability effects may explain the results whereas indirect or choice-based approaches, such as CA and AHP may mitigate such biases (105). While social desirability effects and methodological differences may explain these patterns, it should be emphasized that the present comparison examines different approaches to weighting motivational dimensions rather than contrasting implicit vs. explicit motivation measurement paradigms, which represent fundamentally different theoretical and methodological frameworks (106). Further studies have shown that the method of data collection can significantly influence results (56), likely due to differences in how individuals cognitively process information depending on the evaluation format (107). CA may be particularly useful in simpler decision contexts, while AHP is often better suited for complex, multi-layered decisions (108).

Specifically, AHP and CA represent two distinct methodological approaches: AHP as a multi-criteria decision analysis technique (50) and CA as a stated preference method often implemented through discrete choice experiments (109). Among other differences, AHP uses a decompositional structure where participants evaluate criteria independently while CA takes a compositional approach by asking respondents to evaluate entire profiles or combinations of attributes (110). This structural difference has important implications. For example, Mulye (110) and later Meißner et al. (56) found that although both methods demonstrate similar levels of predictive validity, CA may better simulate realistic decision-making tasks due to its holistic nature. Conversely, AHP may be more suitable when a decision involves more than six criteria because it allows for more structured pairwise comparisons (110).

These findings emphasize that methodological choices can shape how motivational attributes are perceived and weighted, and they should therefore be carefully considered when interpreting results or designing future research in applied selection contexts. As indirect measurements are more externally valid (104), they should be preferred for research questions concerning attribute weightings. However, for results being reliable, it is important to identify the right attributes and to assign them to the right level (104).

## Strengths and limitations

This study offers several methodological and conceptual strengths. First, by comparing three different assessment methods, the study underscores the importance of considering methodological influences when interpreting results. The integration of both CA and AHP provided a valuable complement to direct rating methods, such as CSP, helping to mitigate potential biases such as social desirability and enabling more realistic, decision-oriented evaluations. Additionally, the inclusion of teachers as a control group provided some contextual support for the assumption that domain expertise influences evaluation patterns, as evidenced by the more consistent responses among scouts and recruiters compared to teachers (see Supplementary Material), though this comparison was not a primary focus of the current study.

Limitations should also be noted. One limitation concerns the unequal group sizes, which may have influenced the variability of responses and statistical power across comparisons, however, group sizes were preregistered with the aim of recruiting a maximum possible sample size. Additionally, the gender distribution (24% female scouts and 62% female recruiters) may have an influence on the results due to gender effects. However, the group size as well as gender distribution likely reflect real-world distributions, particularly the substantially higher number of recruiters compared to scouts. Additionally, for the teacher sample, it was not possible to determine whether participants had a background in physical education, which may have introduced additional variability. Furthermore, the exact nature of the jobs for which recruiters were selecting candidates was not specified, potentially limiting the generalizability of the findings to specific occupational roles.

## Practical implications and future research directions

### Practical implication

The findings of this study suggest that motivation should not be treated as a singular construct in applied selection settings. Decision-makers appear to differentiate between distinct types of motivation, with dimensions such as fear of failure gaining particular relevance in the sports context and task orientation in the business context. This differentiation has several implications for improving selection practices.

Decision-makers in selection often rely on intuitive judgements without consciously articulating their evaluation heuristics (48). Making such patterns explicit creates opportunities for evidence-based dialogue about whether current emphases align with organizational goals and empirical evidence. Especially fear of failure's prominence in practice and in research (86, 87) identifies a clear intervention target. For athletes with high fear of failure, targeted psychological interventions could be implemented before selection decisions. If effective, such interventions could expand talent pools by making technically strong but psychologically vulnerable candidates more selection-viable, reducing false-negative selection errors.

The demonstrated expertise effect, i.e., scouts and recruiters showed more consistent evaluation patterns than teachers (see Supplementary Material), reflects the presence of common decision heuristics or shared performance demands across contexts. This indicates that selection competence is learnable (111). Training programs should include dimensional awareness (understanding achievement motivation, goal orientations, self-determination distinctions), bias recognition (75), behavioral indicators (cue perception), and calibration exercises (comparing own weightings against validity evidence).

The observed domain differences further prevent naive cross-domain transfer of assessment approaches without understanding domain-specific emphases. Our findings provide guidance for adaptation: sports’ emphasis on fear of failure and ego orientation could reflect immediate, competitive evaluation structures while business’ task orientation emphasis aligns with long-term, learning-focused demands (37, 93). Organizations can adapt, rather than blindly transplant, weighting dimensions according to their own performance structure.

The results further contribute to the rigor of selection processes by only recording the dimensions that are of interest and are empirically valid. Coaches and practitioners could, for instance, address fear of failure directly to foster more adaptive performance and developmental related behaviours. For example, structured interviews or assessment tools in sports should explicitly probe behavioral indicators of fear of failure rather than relying solely on global motivation ratings. The assessment of fear of failure could be done through questionnaires (112) or observation, as it is done so far. Importantly, valid cues such as reduced risk-taking behavior (113, 114) or adopting avoidance goals (85, 115) need to be precisely observed by coaches (for an overview, see 79) and evaluated for selection. Similarly, selection and subsequent development strategies in corporate environments should adopt a more nuanced approach by combining self-reported motivational profiles with systematic behavioral assessments (e.g., work samples or training-related observations) to reduce social desirability bias.

### Methodological considerations

The observed differences between assessment methods underscore the importance of methodological rigor when measuring preferences for psychological constructs such as motivational dimensions. The constant sum procedure, while simple and direct, appears vulnerable to social desirability effects (105). Our results could be consistent with this, particularly in inflating ratings of intrinsic motivation. This reinforces the need to rely more heavily on more realistic techniques such as AHP or CA in applied research and practice. While more resource-intensive, these methods better reflect realistic decision-making processes and reduce common biases (104). The choice of method should be aligned with the research question, task complexity, and prior methodological recommendations, with awareness that different methods may yield different insights into explicit preferences vs. implicit decision drivers.

### Theoretical implications and future research

This study also contributes to the growing recognition that psychological constructs such as motivation may manifest differently across performance contexts like sports and business: While the dimensions of fear of failure and hope for success are examined intensively in sports (79) there is rare extensive literature on this topic in the economic context to date. However, meaningful overlaps, such as shared emphasis on certain motivational dimensions, indicate potential for cross-contextual comparison.

By comparing two domains, the observed weighting patterns could be evaluated in terms of their generality or contextual specificity. Including business recruitment as a comparison domain therefore strengthens the interpretability of the findings and highlights the importance of cross-domain research for understanding selection heuristics. Future research may extend this comparative approach to additional performance contexts, such as music or education contexts, and combine weighting analyses with person-oriented models to further bridge the gap between selection practice and performance prediction.

By extending this research future work can build a comprehensive evidence base of selection practices that integrate what decision-makers currently emphasize, what actually predicts success, and how both practice and outcomes can be optimized through targeted interventions.

While practitioners often evaluate candidates holistically, understanding which dimensions constitute that “Gestalt” (e.g., 9) represents a necessary first step. Consistently, highly-intrinsically achievement-oriented players have been found to have the highest potential for success (9, 22, 116). Their optimal profile aligns with dimensions we found to be most emphasized by scouts, suggesting that experienced scouts may implicitly recognize these configurations even when unable to articulate the underlying dimension structure (48). For business contexts, profile research should investigate whether similar configurations emerge adapted to different performance structures. While motivational profiles may be particularly informative for predicting performance and development (16, 117), weighting analyses address a complementary question on which aspects of motivation decision-makers actually attend to when making selection judgments. In applied contexts, these implicit weightings, rather than theoretically optimal profiles, often determine selection outcomes (118). Integrating both perspectives, a mechanistic and relational view, may therefore help to identify potential mismatches between predictive validity and actual selection practice (119).

## Conclusion

At first glance, there appear to be clear similarities between selection in sports and in business. Both contexts seem to share a similar view, particularly regarding the importance of motivation. Upon closer inspection, however, it becomes apparent that differences in the importance of various dimensions of motivation exist, although high levels of intrinsic motivation and low levels of fear of failure seem to be important for both. While fear of failure is attributed even greater importance in the sports context, a higher level of task orientation appears to be considered more important in the business context. Possible reasons for this could be the different performance assessments between the contexts: while in sports, performance is shown at a clearly defined point in time and evaluated immediately, job performance is shown over a longer period of time with a focus on the processing of the task. In addition, the differences between recruiters and scouts show that expertise seems to play a role in the assessment of individuals. Also, assessment methods show different results. Based on the method used, results should therefore be interpreted with caution due to the impact the method has. In conclusion, the training of recruiters and scouts should focus on differentiated psychological diagnostics in order to achieve the most valid selection possible. Future research should therefore emphasize on differentiated motivational constructs as well as their individual validity for future success.
