# Correction: The role of motivation in talent selection in sports: insights from a comparison with personnel selection in business

**DOI:** 10.3389/fspor.2026.1702193

**Published:** 2026-02-18

**Authors:** Birte Brinkmöller, Dennis Dreiskämper, Leon Brüggemann, Oliver Höner, Bernd Strauss

**Affiliations:** 1 Department of Sport and Exercise Psychology, Institute of Sport and Exercise Sciences, University of Münster, Münster, Germany; 2 Department of Sports Psychology, Institute for Sport and Sport Science, Dortmund University, Dortmund, Germany; 3 Department of Sport Psychology and Research Methods, Institute of Sports Science, Eberhard Karls University of Tübingen, Tübingen, Germany

Phase 1 of the Registered Report has been replaced with Phase 2.

The original article has been updated.

