# Supplementary material for: The role of motivation in talent selection in sports: insights from a comparison with personnel selection in business
Source: Front Sports Act Living. 2024 Dec 2;6:1463910. doi: 10.3389/fspor.2024.1463910 (PMC11646711; doi:10.3389/fspor.2024.1463910)
Supplement: Supplementary file 1 [file Datasheet1.pdf]

Supplementary Material

Table of Contents

Supplementary Material 1
List of Abbreviations........ *5*
Table S1. Frequency Table of Sports Type of Scouts/Coaches 6
RQ 1. Importance of Motivation in Selection Contexts........ *7*
Figure S1. Average Preference per level (overall) 7
Figure S2. Average Preference per level (sports) 7
Figure S3 Average Preference per level (business) 8
Figure S4. Average Preference per level (Teachers) 8
RQ1 – Subgroup Ranking Orders – Analytical Hierarchy Process *9*
Figure S5. Scouts/Coaches Subgroup Ranking Orders (relative importance) 9
Figure S6. Recruiters Subgroup Ranking Orders (relative importance) 9
Figure S7. Teachers Subgroup Ranking Orders (relative importance)........ 10
RQ 1 – Constant Sum Procedure........ *10*
Figure S8. Scouts/Coaches Subgroup Ranking Orders (relative importance) 10
Figure S9. Recruiters Subgroup Ranking Orders (relative importance) 11
Figure S10. Teachers Subgroup Ranking Orders (relative importance)........ 11
RQ 2.1 Subgroup AMCE and Average Preferences for Level - CJ *12*
Figure S11. Subgroup AMCE 12
Figure S12. Average Preference per Level for Sports 12
Figure S13. Average Preference per Level for Business 13
RQ 2.1 AHP........ *14*
Table S2. Differences between sports and business 14
RQ 2.1 CSP *14*
Table S3. Differences between sports and business 14
RQ 2.2 Differences between Decision Makers - CJ........ *15*
Table S4. Observed Frequencies for Athletes Rated by Recruiters vs. Scouts 15
Table S5. Expected Frequencies for Athletes Rated by Recruiters vs. Scouts........ 15
Table S6. Observed Frequencies for Applicants Rated by Recruiters vs. Scouts 16
Table S7. Expected Frequencies for Applicants Rated by Recruiters vs. Scouts........ 16
Table S8. Chi^2-Test for Athletes Rated by Scouts vs. Teachers 16
Table S9. Observed Frequencies for Athletes Rated by Scouts vs. Teachers........ 17

Table S10. Expected Frequencies for Athletes Rated by Scouts vs. Teachers 17
Table S11. Chi2-Test for Applicants Rated by Scouts vs. Teachers 18
Table S12. Observed Frequencies for Applicants Rated by Scouts vs. Teachers 18
Table S13. Expected Frequencies for Applicants Rated by Scouts vs. Teachers 19
Table S14. Chi^2-Test for Athletes Rated by Recruiters vs. Teachers 19
Table S15. Observed Frequencies for Athletes Rated by Recruiters vs. Teachers 20
Table S16. Expected Frequencies for Athletes Rated by Recruiters vs. Teachers 20
Table S17. Chi^2-Test for Applicants Rated by Recruiters vs. Teachers 20
Table S18. Observed Frequencies for Applicants Rated by Recruiters vs. Teachers 21
Table S19. Expected Frequencies for Applicants Rated by Recruiters vs. Teachers 21
RQ 2.2 Differences between Decision Makers – AHP *23*
Table 20. Chi2-Test for Athletes Rated by Scouts vs. Recruiters 23
Table S21. Observed Frequencies for Athletes Rated by Recruiters vs. Scouts 23
Table S22. Expected Frequencies for Athletes Rated by Recruiters vs. Scouts 23
Table S23. Chi2-Test for Applicants Rated by Scouts vs. Recruiters 24
Table S24. Observed Frequencies for Applicants Rated by Recruiters vs. Scouts 24
Table S25. Expected Frequencies for Applicants Rated by Recruiters vs. Scouts 25
Table S26. Chi2-Test for Athletes Rated by Scouts vs. Teachers 25
Table S27. Observed Frequencies for Athletes Rated by Scouts vs. Teachers 25
Table S28. Expected Frequencies for Athletes Rated by Scouts vs. Teachers 26
Table S29. Chi^2-Test for Applicants Rated by Scouts vs. Teachers 26
Table S30. Observed Frequencies for Applicants Rated by Scouts vs. Teachers 27
Table S31. Expected Frequencies for Applicants Rated by Scouts vs. Teachers 27
Table S32. Chi^2-Test for Athletes Rated by Recruiters vs. Teachers 27
Table S33. Observed Frequencies for Athletes Rated by Recruiters vs. Teachers 28
Table S34. Expected Frequencies for Athletes Rated by Recruiters vs. Teachers 28
Table S35. Chi^2-Test for Applicants Rated by Recruiters vs. Teachers 29
Table S36. Observed Frequencies for Applicants Rated by Recruiters vs. Teachers 29
Table S37. Expected Frequencies for Applicants Rated by Recruiters vs. Teachers 29
Table S38. Chi^2-Test for Athletes Rated by Scouts vs. Recruiters 30
Table S39. Observed Frequencies for Athletes Rated by Recruiters vs. Scouts 30
Table S40. Expected Frequencies for Athletes Rated by Recruiters vs. Scouts 31
Table S41. Chi^2-Test for Applicants Rated by Scouts vs. Recruiters 31
Table S42. Observed Frequencies for Applicants Rated by Recruiters vs. Scouts 32
Table S43. Expected Frequencies for Applicants Rated by Recruiters vs. Scouts 32
Table S44. Chi^2-Test for Athletes Rated by Scouts vs. Teachers 32

Table S45. Observed Frequencies for Athletes Rated by Scouts vs. Teachers 33
Table S46. Expected Frequencies for Athletes Rated by Scouts vs. Teachers 33
Table S47. Chi^2-Test for Applicants Rated by Scouts vs. Teachers 34
Table S48. Observed Frequencies for Applicants Rated by Scouts vs. Teachers 34
Table S49. Expected Frequencies for Applicants Rated by Scouts vs. Teachers 35
Table S50. Chi^2-Test for Athletes Rated by Recruiters vs. Teachers 35
Table S51. Observed Frequencies for Athletes Rated by Recruiters vs. Teachers 35
Table S52. Expected Frequencies for Athletes Rated by Recruiters vs. Teachers 36
Table S53. Chi^2-Test for Applicants Rated by Recruiters vs. Teachers 36
Table S54. Observed Frequencies for Applicants Rated by Recruiters vs. Teachers 37
Table S55. Expected Frequencies for Applicants Rated by Recruiters vs. Teachers 37
RQ 2.3 Differences Between Target Groups – CJ 37
Table S56. Chi^2-Test for Recruiters rating Athletes vs. Applicants 37
Table S57. Observed Frequencies for Varying Expertise Recruiters Rating Applicants vs. Athletes 38
Table S58. Expected Frequencies for Varying Expertise Recruiters Rating Applicants vs. Athletes 38
Table S59. Chi^2-Test for Scouts rating Athletes vs. Applicants 39
Table S60. Observed Frequencies for Varying Expertise Scouts Rating Applicants vs. Athletes 39
Table S61. Expected Frequencies for Scouts Rating Applicants vs. Athletes 40
Table S62. Chi^2-Test for Teachers Rating Applicants vs. Athletes 40
Table S63. Observed Frequencies for Teachers Rating Applicants vs. Athletes 41
Table S64. Expected Frequencies for Teachers Rating Applicants vs. Athletes 41
RQ 2.3 Differences Between Target Groups – AHP 42
Table S65. Chi^2-Test for Scouts rating Athletes vs. Applicants 42
Table S66. Observed Frequencies for Varying Expertise Scouts Rating Applicants vs. Athletes 42
Table S67. Expected Frequencies for Varying Expertise Scouts Rating Applicants vs. Athletes 43
Table S68. Chi^2-Test for Recruiters rating Athletes vs. Applicants 43
Table S69. Observed Frequencies for Varying Expertise Recruiters Rating Applicants vs. Athletes 43
Table S70. Expected Frequencies for Recruiters Rating Applicants vs. Athletes 44
Table S71. Chi^2-Test for Teachers Rating Applicants vs. Athletes 44
Table S72. Observed Frequencies for Teachers Rating Applicants vs. Athletes 45
Table S73. Expected Frequencies for Teachers Rating Applicants vs. Athletes 45

RQ 2.3 Differences Between Target Groups – CSP *45*
Table S74. Chi^2-Test for Scouts rating Athletes vs. Applicants 46
Table S75. Observed Frequencies for Varying Expertise Scouts Rating Applicants vs. Athletes 46
Table S76. Expected Frequencies for Varying Expertise Scouts Rating Applicants vs. Athletes 46
Table S77. Chi^2-Test for Recruiters rating Athletes vs. Applicants 47
Table S78. Observed Frequencies for Varying Expertise Recruiters Rating Applicants vs. Athletes 47
Table S79. Expected Frequencies for Recruiters Rating Applicants vs. Athletes 48
Table S80. Chi^2-Test for Teachers Rating Applicants vs. Athletes 48
Table S81. Observed Frequencies for Teachers Rating Applicants vs. Athletes 48
Table S82. Expected Frequencies for Teachers Rating Applicants vs. Athletes 49
RQ 2.4 Differences between Assessment Methods *50*
Table S83. Hope for Success in Athletes 50
Table S84. Hope for Success in Applicants 50
Table S85. Fear of Failure in Athletes 51
Table S86. Fear of Failure in Applicants 51
Table S87. Ego Orientation in Athletes 52
Table S88. Ego Orientation in Applicants 52
Table S89. Task Orientation in Athletes 53
Table S90. Task Orientation in Applicants 53
Table S91. Intrinsic Motivation in Athletes 54
Table S92. Intrinsic Motivation in Applicants 54
Table S93. Extrinsic Motivation in Athletes 55
Table S94. Extrinsic Motivation in Applicants 55

List of Abbreviations

Dimension	Abbreviation
Hope for Success Business	H.B
Hope for Success Sport	H.S
Hope for Success Teacher	H.T
Fear of Failure Business	F.B
Fear of Failure Sport	F.S
Fear of Failure Teacher	F.T
Ego Orientation Business	EO.B
Ego Orientation Sport	EO.S
Ego Orientation Teacher	EO.T
Task Orientation Business	TO.B
Task Orientation Sport	TO.S
Task Orientation Teacher	TO.T
Intrinsic Motivation Business	IM.B
Intrinsic Motivation Sport	IM.S
Intrinsic Motivation Teacher	IM.T
Extrinsic Motivation Business	EM.B
Extrinsic Motivation Sport	EM.S
Extrinsic Motivation Teacher	EM.T

Table S1. Frequency Table of Sports Type of Scouts/Coaches

Sports type	Frequencies
Basketball	4
Ice hockey	1
Field hockey	1
Soccer (female)	1
Soccer (male)	12
Handball	4
Hockey	2
Volleyball	1

RQ 1. Importance of Motivation in Selection Contexts

Figure S1. Average Preference per level (overall)

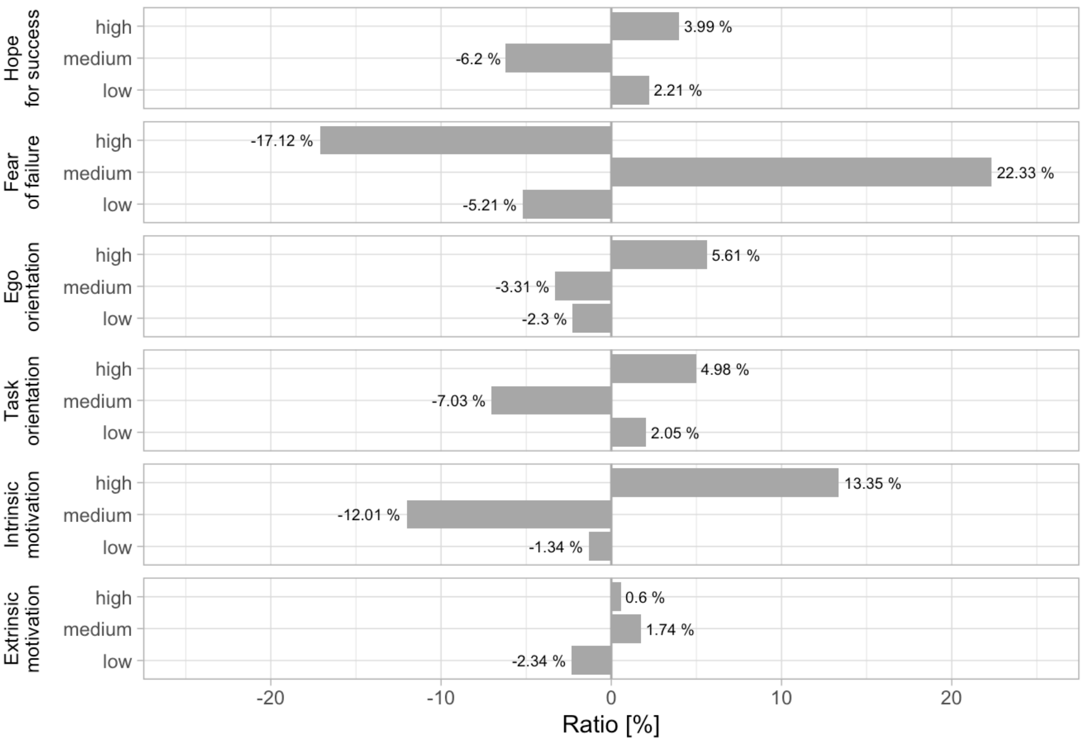

Figure S2. Average Preference per level (sports)

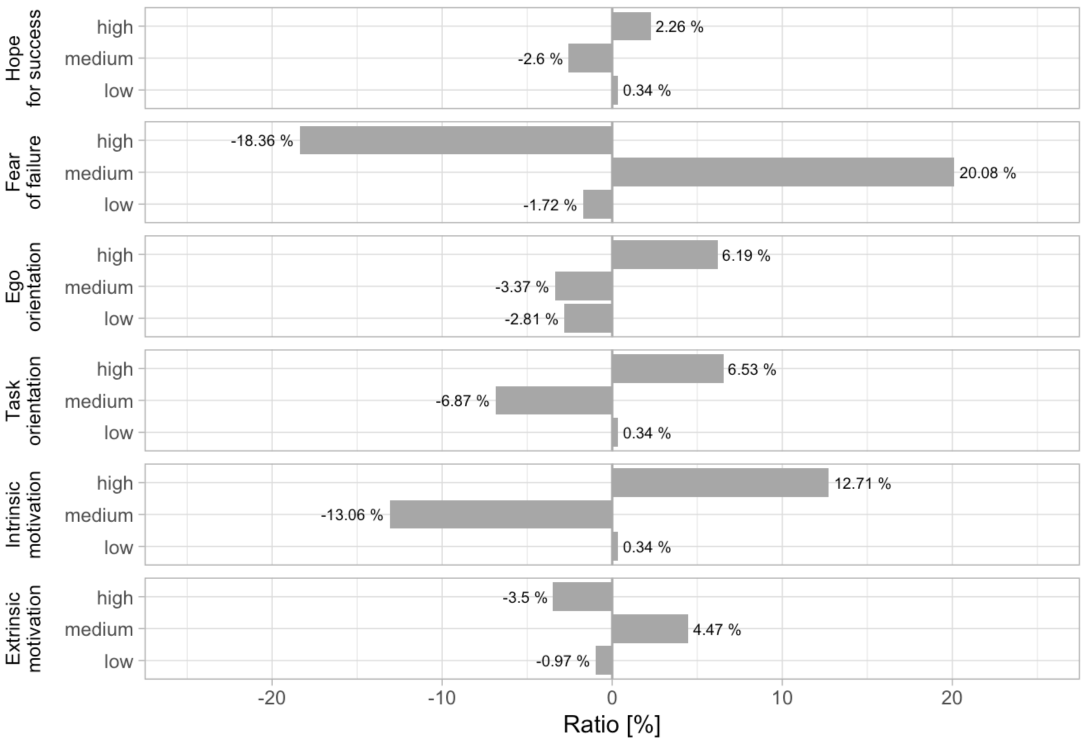

Figure S3 Average Preference per level (business)

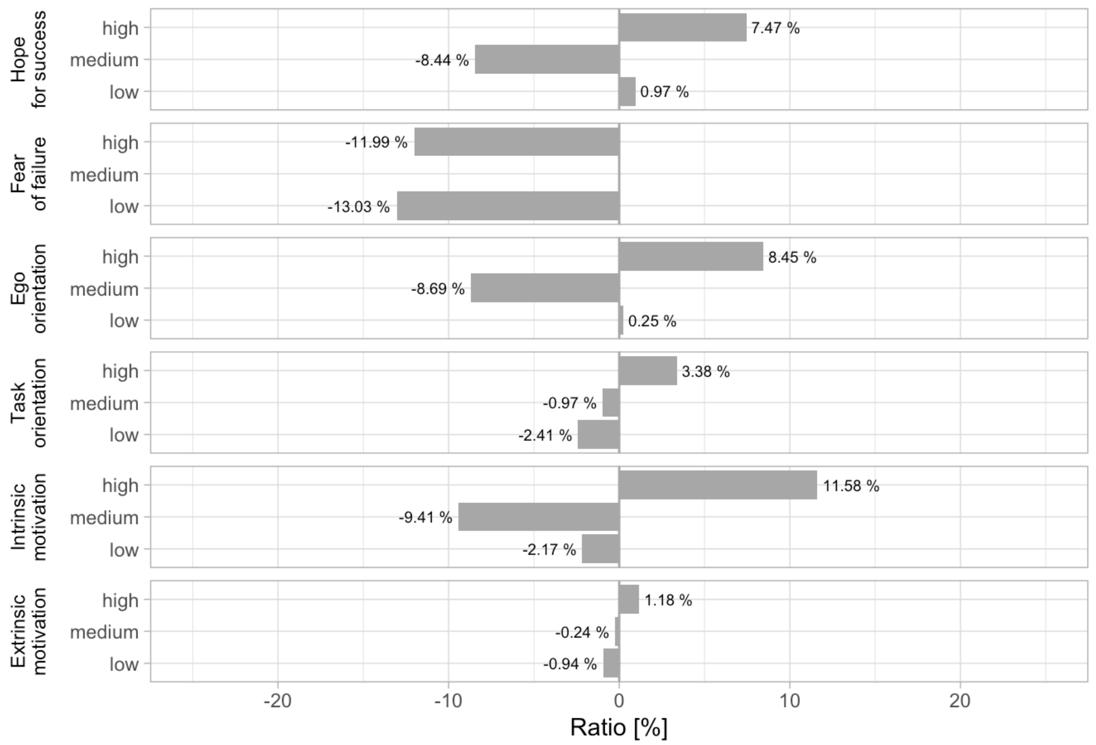

Figure S4. Average Preference per level (Teachers)

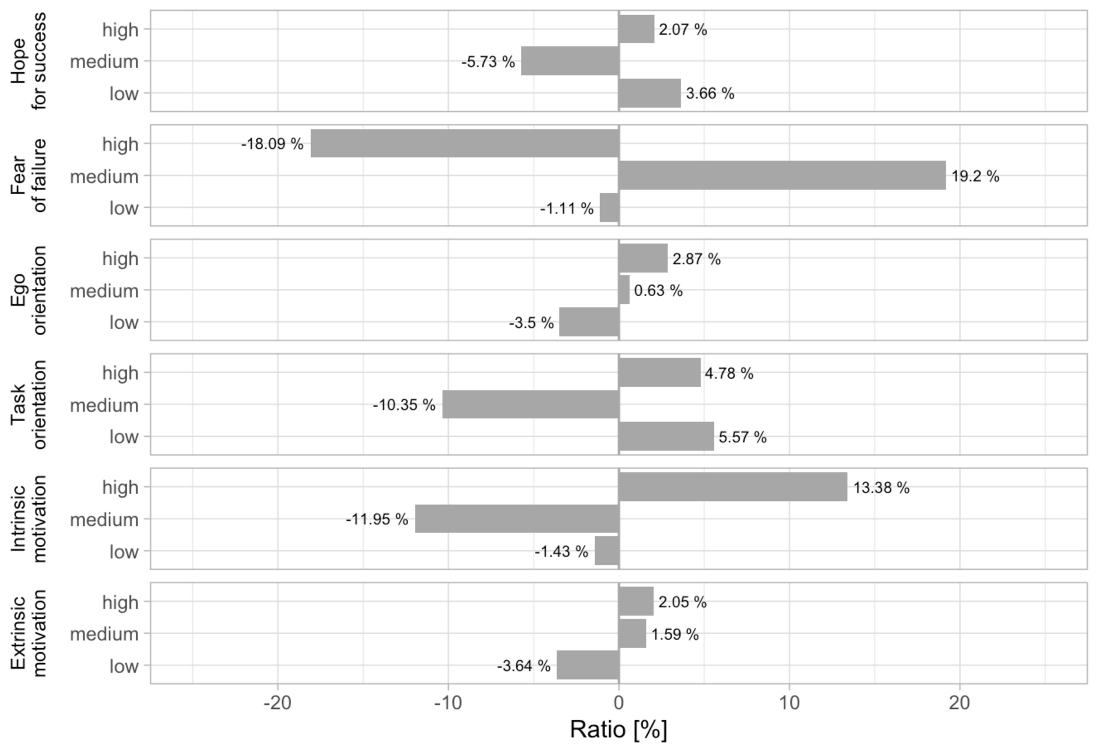

RQ1 – Subgroup Ranking Orders – Analytical Hierarchy Process

Figure S5. Scouts/Coaches Subgroup Ranking Orders (relative importance)

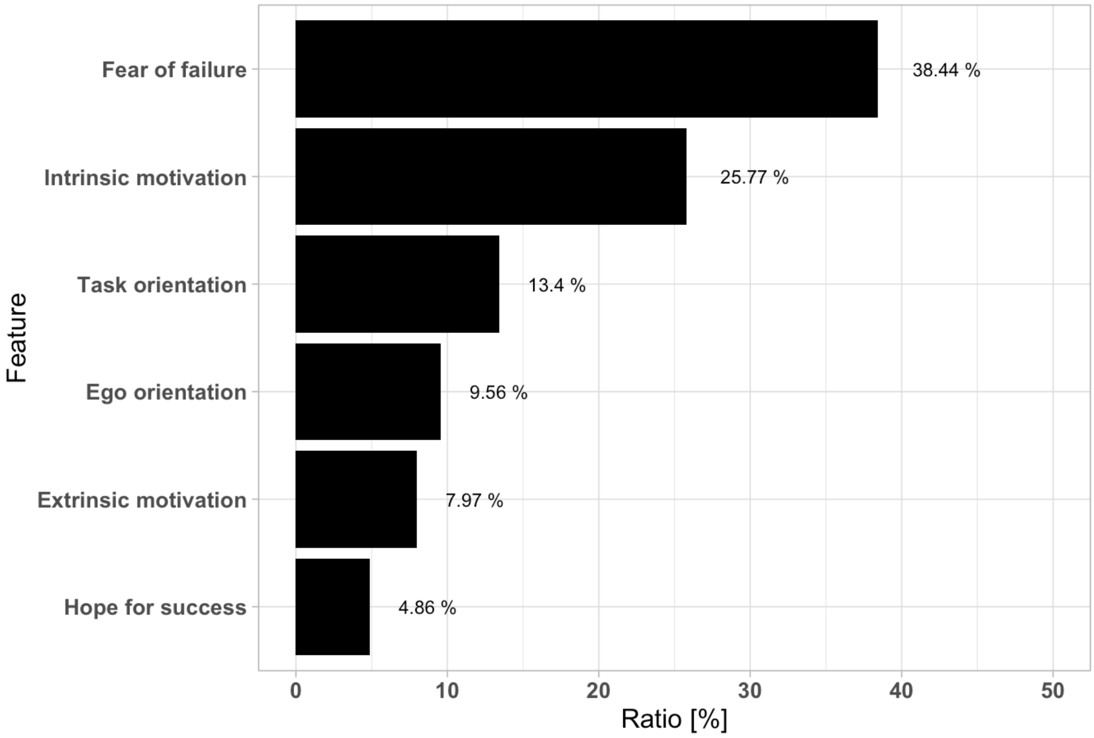

Figure S6. Recruiters Subgroup Ranking Orders (relative importance)

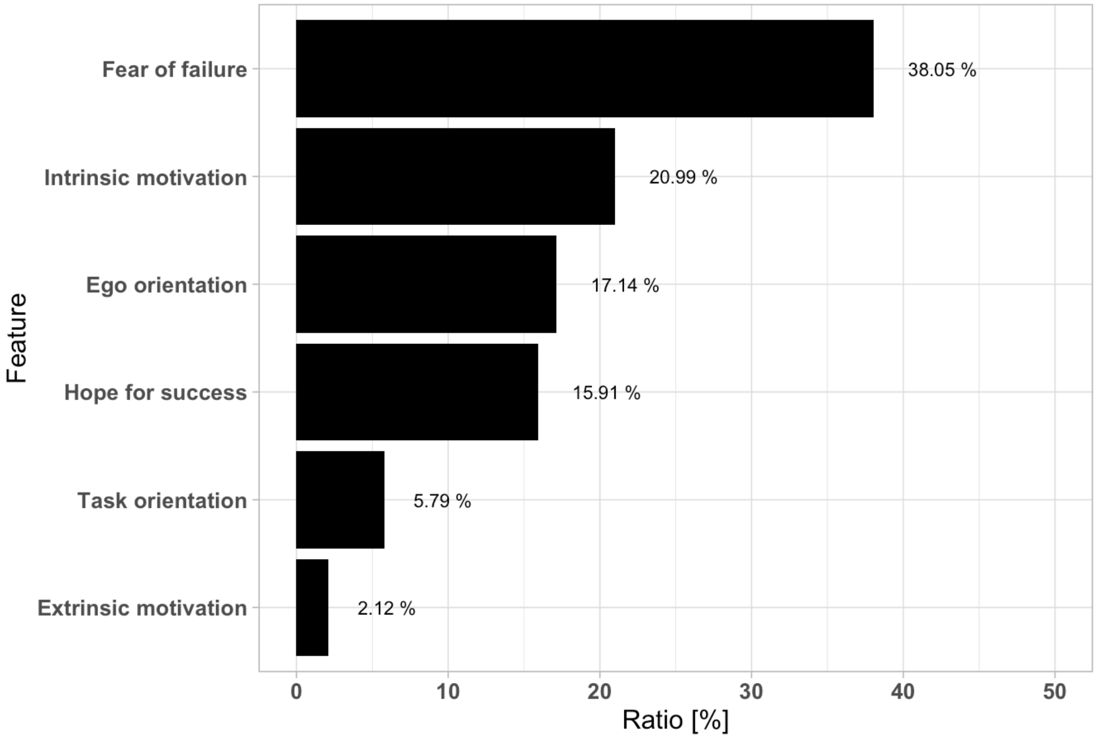

Figure S7. Teachers Subgroup Ranking Orders (relative importance)

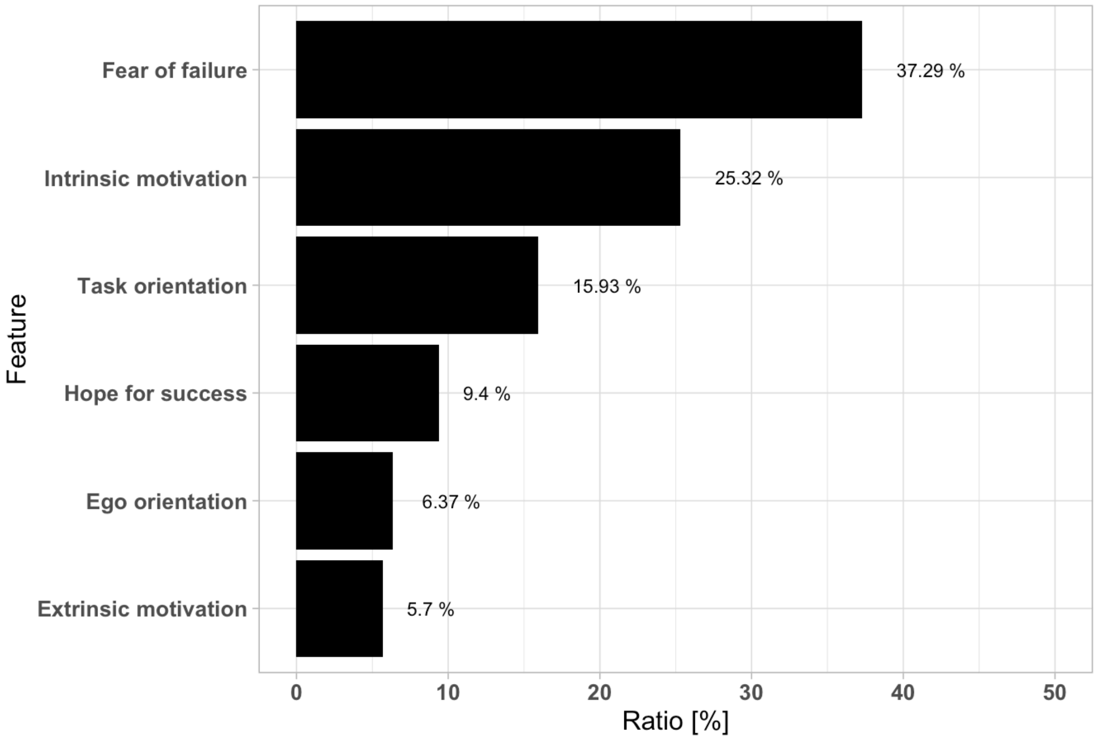

RQ 1 – Constant Sum Procedure

Figure S8. Scouts/Coaches Subgroup Ranking Orders (relative importance)

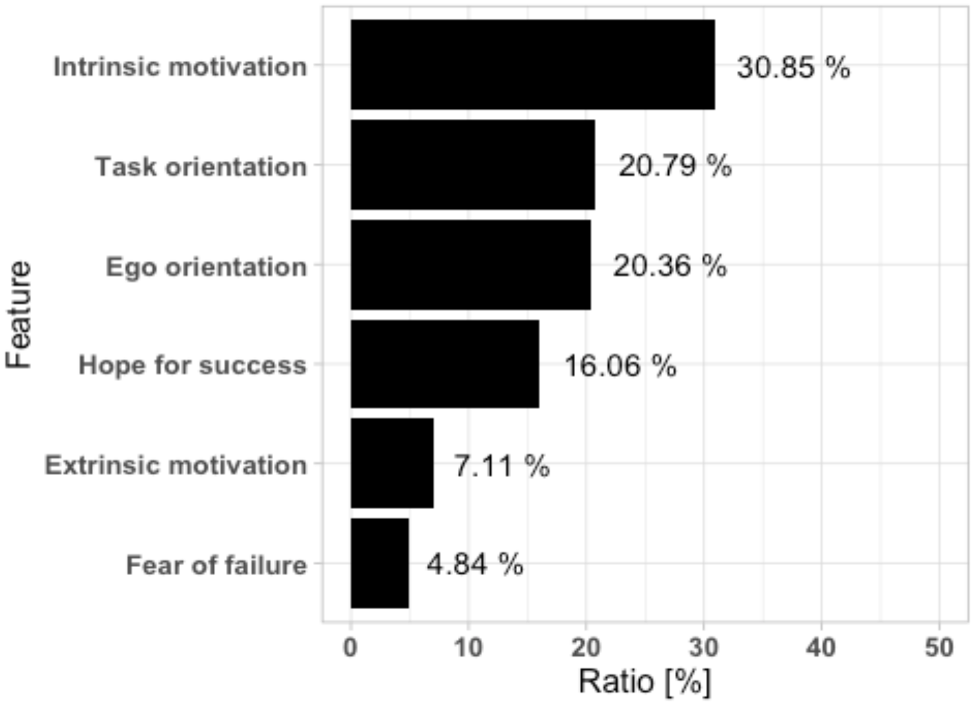

Figure S9. Recruiters Subgroup Ranking Orders (relative importance)

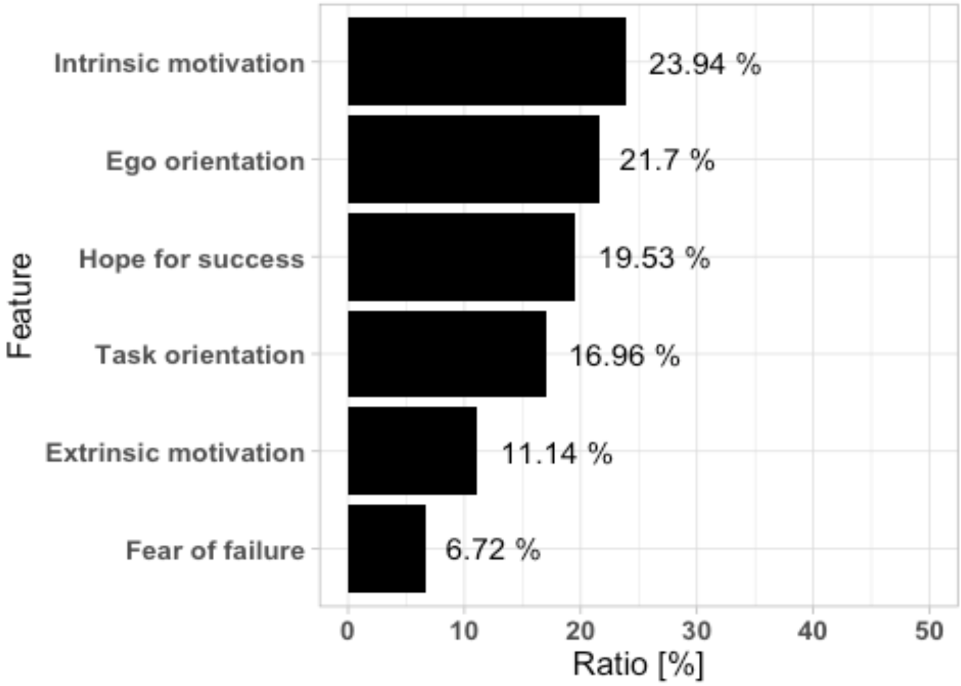

Figure S10. Teachers Subgroup Ranking Orders (relative importance)

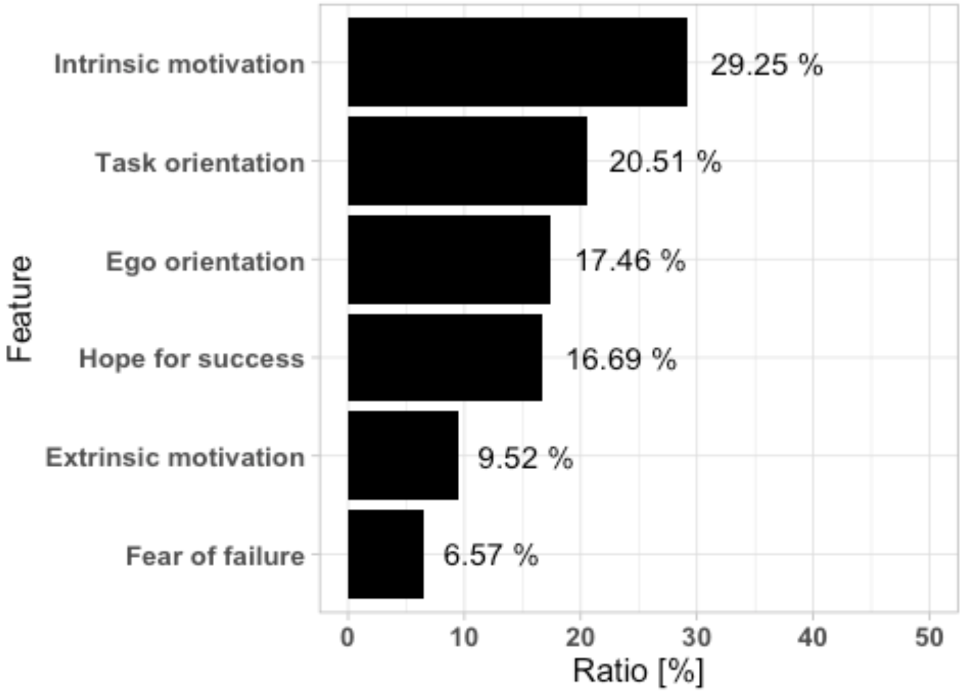

RQ 2.1 Subgroup AMCE and Average Preferences for Level - CJ

Figure S11. Subgroup AMCE

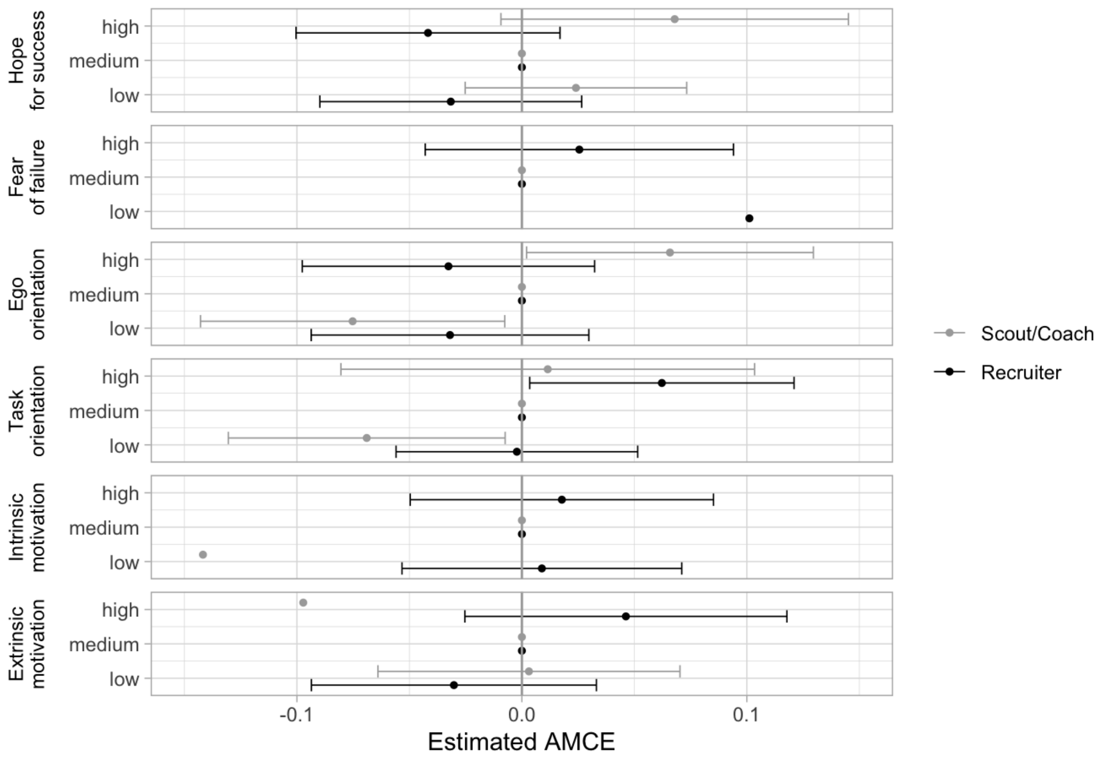

Figure S12. Average Preference per Level for Sports

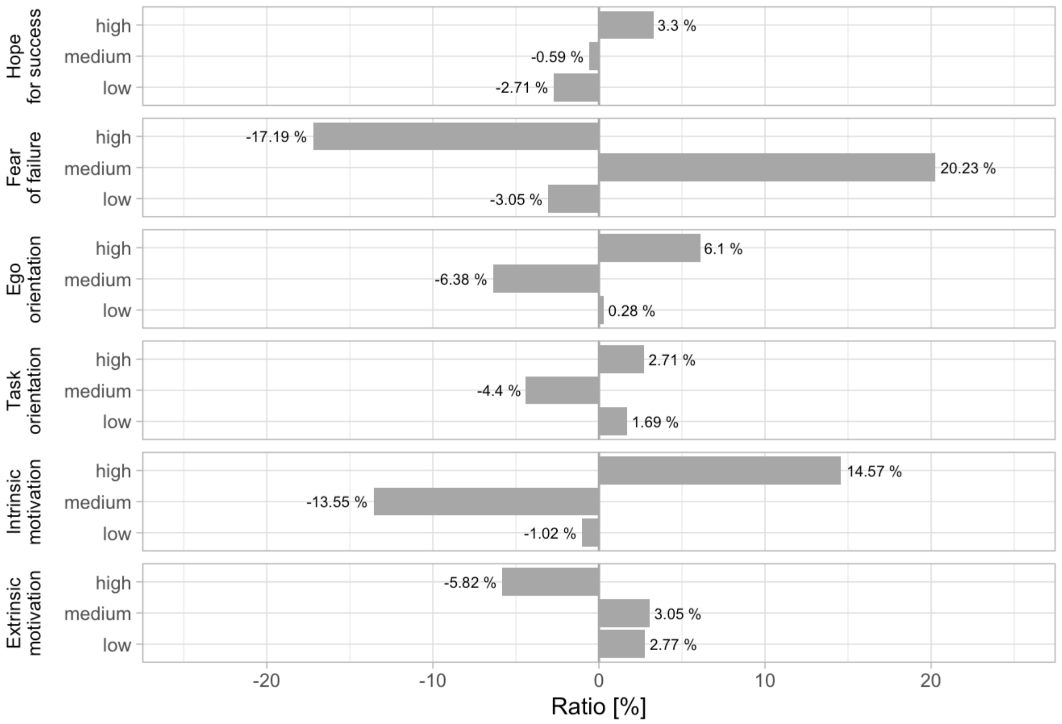

Figure S13. Average Preference per Level for Business

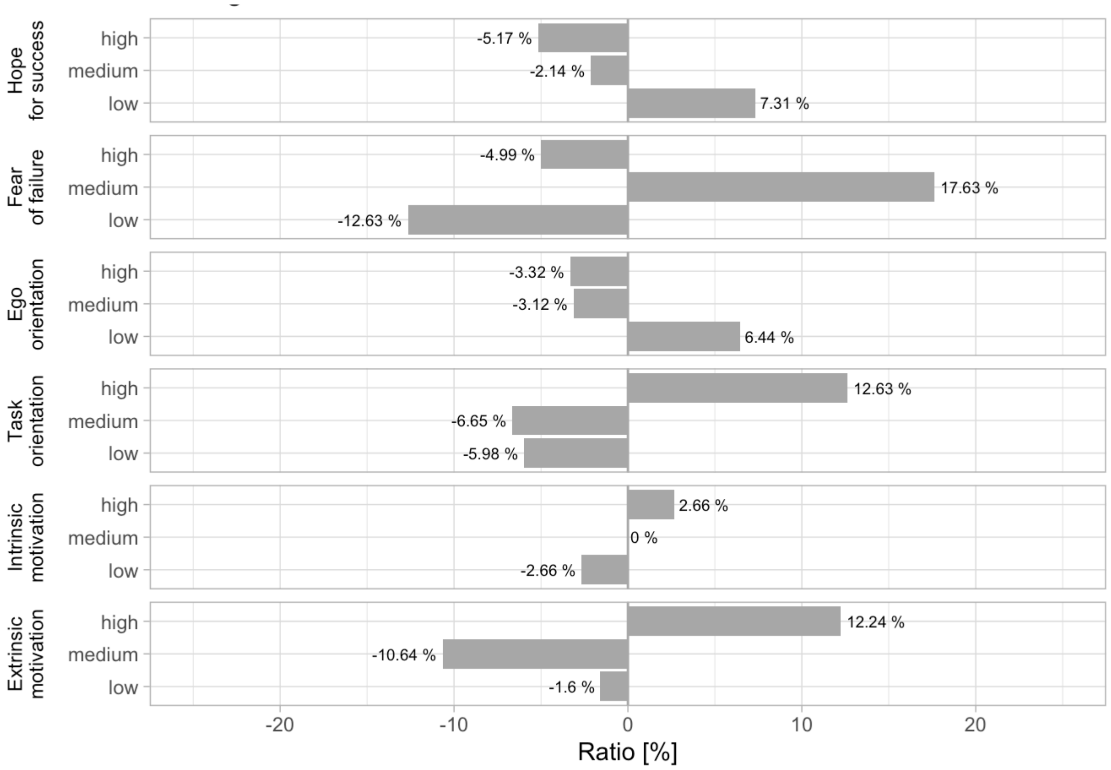

RQ 2.1 AHP

Table S2. Differences between sports and business

Variable	F	η^2	p
Ego Orientation	43.4	0.373	0.00
Extrinsic Motivation	.007	0.00	0.931
Fear of Failure	20.2	0.216	0.00
Hope for Success	2.33	0.031	0.131
Intrinsic Motivation	1.96	0.026	0.166
Task Orientation	33.3	0.313	0.00

RQ 2.1 CSP

Table S3. Differences between sports and business

Variable	F	η^2	p
Ego Orientation	0.218	0.003	0.616
Extrinsic Motivation	3.72	0.046	0.058
Fear of Failure	6.21	0.075	0.015
Hope for Success	0.253	0.003	0.616
Intrinsic Motivation	5.42	0.066	0.022
Task Orientation	0.309	0.004	0.58

RQ 2.2 Differences between Decision Makers - CJ

Table S4. Observed Frequencies for Athletes Rated by Recruiters vs. Scouts

Rank	H.B	H.S	F.B	F.S	EO.B	EO.S	TO.B	TO.S	IM.B	IM.S	EM.B	EM.S
1	9	1	10	16	8	1	5	4	11	6	10	1
2	7	3	10	6	15	8	4	4	7	5	6	4
3	7	4	17	1	8	6	9	4	5	9	7	13
4	10	3	4	2	10	3	13	7	13	5	5	4
5	4	9	3	4	5	8	15	4	5	1	13	4
6	13	9	6	0	4	3	4	6	9	3	9	3

Table S5. Expected Frequencies for Athletes Rated by Recruiters vs. Scouts

Rank	H.B	H.S	F.B	F.S	EO.B	EO.S	TO.B	TO.S	IM.B	IM.S	EM.B	EM.S
1	6.33	3.67	16.46	9.54	5.70	3.30	5.70	3.30	10.76	6.24	6.96	4.04
2	6.33	3.67	10.13	5.87	14.56	8.44	5.06	2.94	7.59	4.41	6.33	3.67
3	6.96	4.04	11.39	6.61	8.86	5.14	8.23	4.77	8.86	5.14	12.66	7.34
4	8.23	4.77	3.80	2.20	8.23	4.77	12.66	7.34	11.39	6.61	5.70	3.30
5	8.23	4.77	4.43	2.57	8.23	4.77	12.03	6.97	3.80	2.20	10.76	6.24
6	13.92	8.08	3.80	2.20	4.43	2.57	6.33	3.67	7.59	4.41	7.59	4.41

Table S6. Observed Frequencies for Applicants Rated by Recruiters vs. Scouts

Rank	H.B	H.S	F.B	F.S	EO.B	EO.S	TO.B	TO.S	IM.B	IM.S	EM.B	EM.S
1	13	7	8	4	10	3	6	8	9	1	9	8
2	5	6	12	9	9	2	6	3	11	4	12	6
3	8	4	6	8	9	6	10	5	12	4	6	4
4	5	3	11	3	7	7	10	6	10	9	7	4
5	10	4	8	3	10	6	7	5	5	6	10	3
6	9	5	5	2	5	5	11	2	3	5	6	4

Table S7. Expected Frequencies for Applicants Rated by Recruiters vs. Scouts

Rank	H.B	H.S	F.B	F.S	EO.B	EO.S	TO.B	TO.S	IM.B	IM.S	EM.B	EM.S
1	12.66	7.34	7.59	4.41	8.23	4.77	8.86	5.14	6.33	3.67	10.76	6.24
2	6.96	4.04	13.29	7.71	6.96	4.04	5.70	3.30	9.49	5.51	11.39	6.61
3	7.59	4.41	8.86	5.14	9.49	5.51	9.49	5.51	10.13	5.87	6.33	3.67
4	5.06	2.94	8.86	5.14	8.86	5.14	10.13	5.87	12.03	6.97	6.96	4.04
5	8.86	5.14	6.96	4.04	10.13	5.87	7.59	4.41	6.96	4.04	8.23	4.77
6	8.86	5.14	4.43	2.57	6.33	3.67	8.23	4.77	5.06	2.94	6.33	3.67

Table S8. Chi²-Test for Athletes Rated by Scouts vs. Teachers

Variable	$\chi 2$	*df*	*Cramer's V*	*p*
Ego Orientation	7.30	NA	0.27	0.201

Variable	*χ2*	*df*	*Cramer's V*	*p*
Extrinsic Motivation	10.88	NA	0.32	0.053
Fear of Failure	6.09	NA	0.24	0.296
Hope for Success	5.14	NA	0.22	0.414
Intrinsic Motivation	6.24	NA	0.25	0.289
Task Orientation	1.32	NA	0.11	0.942

Note. df = NA indicates that Monte-Carlo simulation for Chi-squared was applied

Table S9. Observed Frequencies for Athletes Rated by Scouts vs. Teachers

Rank	H.S	H.T	F.S	F.T	EO.S	EO.T	TO.S	TO.T	IM.S	IM.T	EM.S	EM.T
1	1	6	16	26	1	7	4	15	6	14	1	9
2	3	12	6	15	8	7	4	9	5	17	4	15
3	4	17	1	12	6	15	4	11	9	11	13	12
4	3	11	2	7	3	16	7	17	5	12	4	16
5	9	13	4	11	8	19	4	12	1	13	4	8
6	9	15	0	3	3	10	6	10	3	7	3	14

Table S10. Expected Frequencies for Athletes Rated by Scouts vs. Teachers

Rank	H.S	H.T	F.S	F.T	EO.S	EO.T	TO.S	TO.T	IM.S	IM.T	EM.S	EM.T
1	1.97	5.03	11.83	30.17	2.25	5.75	5.35	13.65	5.63	14.37	2.82	7.18
2	4.22	10.78	5.91	15.09	4.22	10.78	3.66	9.34	6.19	15.81	5.35	13.65

3	5.91	15.09	3.66	9.34	5.91	15.09	4.22	10.78	5.63	14.37	7.04	17.96
4	3.94	10.06	2.53	6.47	5.35	13.65	6.76	17.24	4.79	12.21	5.63	14.37
5	6.19	15.81	4.22	10.78	7.60	19.40	4.50	11.50	3.94	10.06	3.38	8.62
6	6.76	17.24	0.84	2.16	3.66	9.34	4.50	11.50	2.82	7.18	4.79	12.21

Table S11. Chi2-Test for Applicants Rated by Scouts vs. Teachers

Variable	$\chi 2$	*df*	*Cramer's V*	*p*
Ego Orientation	6.18	NA	0.24	0.298
Extrinsic Motivation	2.77	5	0.16	0.735
Fear of Failure	4.96	NA	0.22	0.435
Hope for Success	3.16	NA	0.18	0.691
Intrinsic Motivation	4.29	NA	0.20	0.518
Task Orientation	4.56	NA	0.21	0.486

Note. df = NA indicates that Monte-Carlo simulation for Chi-squared was applied

Table S12. Observed Frequencies for Applicants Rated by Scouts vs. Teachers

Rank	H.S	H.T	F.S	F.T	EO.S	EO.T	TO.S	TO.T	IM.S	IM.T	EM.S	EM.T
1	7	16	4	10	3	17	8	11	1	9	8	15
2	6	17	9	16	2	8	3	16	4	13	6	12
3	4	13	8	12	6	17	5	10	4	14	4	17
4	3	12	3	8	7	7	6	14	9	17	4	14

5	4	11	3	15	6	10	5	12	6	15	3	10
6	5	5	2	13	5	15	2	11	5	6	4	6

Table S13. Expected Frequencies for Applicants Rated by Scouts vs. Teachers

Rank	H.S	H.T	F.S	F.T	EO.S	EO.T	TO.S	TO.T	IM.S	IM.T	EM.S	EM.T
1	6.48	16.52	3.94	10.06	5.63	14.37	5.35	13.65	2.82	7.18	6.48	16.52
2	6.48	16.52	7.04	17.96	2.82	7.18	5.35	13.65	4.79	12.21	5.07	12.93
3	4.79	12.21	5.63	14.37	6.48	16.52	4.22	10.78	5.07	12.93	5.91	15.09
4	4.22	10.78	3.10	7.90	3.94	10.06	5.63	14.37	7.32	18.68	5.07	12.93
5	4.22	10.78	5.07	12.93	4.50	11.50	4.79	12.21	5.91	15.09	3.66	9.34
6	2.82	7.18	4.22	10.78	5.63	14.37	3.66	9.34	3.10	7.90	2.82	7.18

Table S14. Chi^2-Test for Athletes Rated by Recruiters vs. Teachers

Variable	*χ2*	*df*	*Cramer's V*	*p*
Ego Orientation	13.07	5	0.32	0.023*
Extrinsic Motivation	8.96	5	0.27	0.111
Fear of Failure	11.14	5	0.30	0.049*
Hope for Success	6.64	5	0.23	0.249
Intrinsic Motivation	6.21	5	0.22	0.286
Task Orientation	6.15	5	0.22	0.292

Table S15. Observed Frequencies for Athletes Rated by Recruiters vs. Teachers

Rank	H.B	H.T	F.B	F.T	EO.B	EO.T	TO.B	TO.T	IM.B	IM.T	EM.B	EM.T
1	9	6	10	26	8	7	5	15	11	14	10	9
2	7	12	10	15	15	7	4	9	7	17	6	15
3	7	17	17	12	8	15	9	11	5	11	7	12
4	10	11	4	7	10	16	13	17	13	12	5	16
5	4	13	3	11	5	19	15	12	5	13	13	8
6	13	15	6	3	4	10	4	10	9	7	9	14

Table S16. Expected Frequencies for Athletes Rated by Recruiters vs. Teachers

Rank	H.B	H.T	F.B	F.T	EO.B	EO.T	TO.B	TO.T	IM.B	IM.T	EM.B	EM.T
1	6.05	8.95	14.52	21.48	6.05	8.95	8.06	11.94	10.08	14.92	7.66	11.34
2	7.66	11.34	10.08	14.92	8.87	13.13	5.24	7.76	9.68	14.32	8.47	12.53
3	9.68	14.32	11.69	17.31	9.27	13.73	8.06	11.94	6.45	9.55	7.66	11.34
4	8.47	12.53	4.44	6.56	10.48	15.52	12.10	17.90	10.08	14.92	8.47	12.53
5	6.85	10.15	5.65	8.35	9.68	14.32	10.89	16.11	7.26	10.74	8.47	12.53
6	11.29	16.71	3.63	5.37	5.65	8.35	5.65	8.35	6.45	9.55	9.27	13.73

Table S17. Chi^2-Test for Applicants Rated by Recruiters vs. Teachers

Variable	$\chi2$	*df*	*Cramer's V*	*p*
Ego Orientation	4.87	5	0.20	0.432

Variable	$\chi 2$	*df*	*Cramer's V*	*p*
Extrinsic Motivation	4.62	5	0.19	0.464
Fear of Failure	4.48	5	0.19	0.483
Hope for Success	7.76	5	0.25	0.170
Intrinsic Motivation	3.63	5	0.17	0.604
Task Orientation	3.48	5	0.17	0.626

Table S18. Observed Frequencies for Applicants Rated by Recruiters vs. Teachers

Rank	H.B	H.T	F.B	F.T	EO.B	EO.T	TO.B	TO.T	IM.B	IM.T	EM.B	EM.T
1	13	16	8	10	10	17	6	11	9	9	9	15
2	5	17	12	16	9	8	6	16	11	13	12	12
3	8	13	6	12	9	17	10	10	12	14	6	17
4	5	12	11	8	7	7	10	14	10	17	7	14
5	10	11	8	15	10	10	7	12	5	15	10	10
6	9	5	5	13	5	15	11	11	3	6	6	6

Table S19. Expected Frequencies for Applicants Rated by Recruiters vs. Teachers

Rank	H.B	H.T	F.B	F.T	EO.B	EO.T	TO.B	TO.T	IM.B	IM.T	EM.B	EM.T
1	11.69	17.31	7.26	10.74	10.89	16.11	6.85	10.15	7.26	10.74	9.68	14.32
2	8.87	13.13	11.29	16.71	6.85	10.15	8.87	13.13	9.68	14.32	9.68	14.32
3	8.47	12.53	7.26	10.74	10.48	15.52	8.06	11.94	10.48	15.52	9.27	13.73
4	6.85	10.15	7.66	11.34	5.65	8.35	9.68	14.32	10.89	16.11	8.47	12.53

5	8.47	12.53	9.27	13.73	8.06	11.94	7.66	11.34	8.06	11.94	8.06	11.94
6	5.65	8.35	7.26	10.74	8.06	11.94	8.87	13.13	3.63	5.37	4.84	7.16

RQ 2.2 Differences between Decision Makers – AHP

Table 20. Chi2-Test for Athletes Rated by Scouts vs. Recruiters

Variable	$\chi 2$	*df*	*Cramer's V*	*p*
Ego Orientation	2.78	NA	0.19	.760
Extrinsic Motivation	4.42	NA	0.24	.534
Fear of Failure	7.15	NA	0.31	.212
Hope for Success	2.73	NA	0.19	.757
Intrinsic Motivation	6.38	NA	0.29	.274
Task Orientation	13.99	NA	0.43	.011*

Note. df = NA indicates that Monte-Carlo simulation for Chi-squared was applied

Table S21. Observed Frequencies for Athletes Rated by Recruiters vs. Scouts

Rank	H.B	H.S	F.B	F.S	EO.B	EO.S	TO.B	TO.S	IM.B	IM.S	EM.B	EM.S
1	6	2	11	11	9	4	6	4	18	5	1	1
2	6	4	11	9	11	4	1	1	14	10	5	1
3	10	7	8	3	7	7	12	1	9	6	4	1
4	9	8	5	2	13	9	8	4	7	3	5	1
5	10	4	10	1	6	2	10	15	1	1	12	5
6	8	2	4	1	3	1	12	2	0	2	22	18

Table S22. Expected Frequencies for Athletes Rated by Recruiters vs. Scouts

Rank	H.B	H.S	F.B	F.S	EO.B	EO.S	TO.B	TO.S	IM.B	IM.S	EM.B	EM.S
1	5.16	2.84	14.18	7.82	8.38	4.62	6.45	3.55	14.83	8.17	1.29	0.71
2	6.45	3.55	12.89	7.11	9.67	5.33	1.29	0.71	15.47	8.53	3.87	2.13

3	10.96	6.04	7.09	3.91	9.03	4.97	8.38	4.62	9.67	5.33	3.22	1.78
4	10.96	6.04	4.51	2.49	14.18	7.82	7.74	4.26	6.45	3.55	3.87	2.13
5	9.03	4.97	7.09	3.91	5.16	2.84	16.12	8.88	1.29	0.71	10.96	6.04
6	6.45	3.55	3.22	1.78	2.58	1.42	9.03	4.97	1.29	0.71	25.79	14.21

Table S23. Chi2-Test for Applicants Rated by Scouts vs. Recruiters

Variable	*χ2*	*df*	*Cramer's V*	*p*
Ego Orientation	5.44	NA	0.27	.388
Extrinsic Motivation	10.74	NA	0.38	.03*
Fear of Failure	7.45	NA	0.32	.192
Hope for Success	3.26	NA	0.21	.687
Intrinsic Motivation	6.45	NA	0.29	.167
Task Orientation	7.27	NA	0.31	.120

Note. df = NA indicates that Monte-Carlo simulation for Chi-squared was applied

Table S24. Observed Frequencies for Applicants Rated by Recruiters vs. Scouts

Rank	H.B	H.S	F.B	F.S	EO.B	EO.S	TO.B	TO.S	IM.B	IM.S	EM.B	EM.S
1	7	3	5	1	2	3	15	6	18	15	1	0
2	6	3	8	5	0	1	21	11	14	5	0	1
3	16	8	21	6	2	0	3	7	6	5	0	3
4	12	11	10	9	3	1	6	3	7	0	9	1
5	6	1	3	3	15	11	3	0	3	2	19	12

Table S25. Expected Frequencies for Applicants Rated by Recruiters vs. Scouts

Rank	H.B	H.S	F.B	F.S	EO.B	EO.S	TO.B	TO.S	IM.B	IM.S	EM.B	EM.S
1	6.40	3.60	3.84	2.16	3.20	1.80	13.44	7.56	21.12	11.88	0.64	0.36
2	5.76	3.24	8.32	4.68	0.64	0.36	20.48	11.52	12.16	6.84	0.64	0.36
3	15.36	8.64	17.28	9.72	1.28	0.72	6.40	3.60	7.04	3.96	1.92	1.08
4	14.72	8.28	12.16	6.84	2.56	1.44	5.76	3.24	4.48	2.52	6.40	3.60
5	4.48	2.52	3.84	2.16	16.64	9.36	1.92	1.08	3.20	1.80	19.84	11.16

Table S26. Chi2-Test for Athletes Rated by Scouts vs. Teachers

Variable	$\chi 2$	*df*	*Cramer's V*	*p*
Ego Orientation	8.91	NA	0.30	0.101
Extrinsic Motivation	4.48	NA	0.22	0.468
Fear of Failure	4.35	NA	0.21	0.499
Hope for Success	2.40	NA	0.16	0.806
Intrinsic Motivation	6.32	NA	0.26	0.282
Task Orientation	12.47	NA	0.36	0.026*

Note. df = NA indicates that Monte-Carlo simulation for Chi-squared was applied

Table S27. Observed Frequencies for Athletes Rated by Scouts vs. Teachers

Rank	H.S	H.T	F.S	F.T	EO.S	EO.T	TO.S	TO.T	IM.S	IM.T	EM.S	EM.T
1	2	7	11	26	4	25	4	2	5	17	1	1
2	4	11	9	16	4	14	1	4	10	22	1	3
3	7	9	3	20	7	14	1	3	6	14	1	1
4	8	25	2	5	9	11	4	9	3	11	1	7

5	4	11	1	1	2	5	15	25	1	5	5	23
6	2	6	1	1	1	0	2	26	2	0	18	34

Table S28. Expected Frequencies for Athletes Rated by Scouts vs. Teachers

Rank	H.S	H.T	F.S	F.T	EO.S	EO.T	TO.S	TO.T	IM.S	IM.T	EM.S	EM.T
1	2.53	6.47	10.41	26.59	8.16	20.84	1.69	4.31	6.19	15.81	0.56	1.44
2	4.22	10.78	7.03	17.97	5.06	12.94	1.41	3.59	9.00	23.00	1.12	2.88
3	4.50	11.50	6.47	16.53	5.91	15.09	1.12	2.88	5.62	14.38	0.56	1.44
4	9.28	23.72	1.97	5.03	5.62	14.38	3.66	9.34	3.94	10.06	2.25	5.75
5	4.22	10.78	0.56	1.44	1.97	5.03	11.25	28.75	1.69	4.31	7.88	20.12
6	2.25	5.75	0.56	1.44	0.28	0.72	7.88	20.12	0.56	1.44	14.62	37.38

Table S29. Chi^2-Test for Applicants Rated by Scouts vs. Teachers

Variable	$\chi 2$	*df*	*Cramer's V*	*p*
Ego Orientation	6.96	NA	0.27	0.131
Extrinsic Motivation	4.04	NA	0.20	0.574
Fear of Failure	4.24	NA	0.21	0.536
Hope for Success	6.03	NA	0.25	0.314
Intrinsic Motivation	5.26	NA	0.23	0.395
Task Orientation	3.00	NA	0.18	0.589

Note. df = NA indicates that Monte-Carlo simulation for Chi-squared was applied

Table S30. Observed Frequencies for Applicants Rated by Scouts vs. Teachers

Rank	H.S	H.T	F.S	F.T	EO.S	EO.T	TO.S	TO.T	IM.S	IM.T	EM.S	EM.T
1	3	7	1	12	3	3	6	22	15	36	0	3
2	3	14	5	8	1	3	11	25	5	19	1	2
3	8	24	6	14	0	0	7	11	5	9	3	5
4	1	7	3	9	11	14	0	3	2	1	12	38
5	11	18	9	23	1	4	3	9	0	4	1	6
6	1	0	3	4	11	46	0	0	0	1	10	16

Table S31. Expected Frequencies for Applicants Rated by Scouts vs. Teachers

Rank	H.S	H.T	F.S	F.T	EO.S	EO.T	TO.S	TO.T	IM.S	IM.T	EM.S	EM.T
1	2.78	7.22	3.62	9.38	1.67	4.33	7.79	20.21	14.20	36.80	0.84	2.16
2	4.73	12.27	3.62	9.38	1.11	2.89	10.02	25.98	6.68	17.32	0.84	2.16
3	8.91	23.09	5.57	14.43	0.00	0.00	5.01	12.99	3.90	10.10	2.23	5.77
4	2.23	5.77	3.34	8.66	6.96	18.04	0.84	2.16	0.84	2.16	13.92	36.08
5	8.07	20.93	8.91	23.09	1.39	3.61	3.34	8.66	1.11	2.89	1.95	5.05
6	0.28	0.72	1.95	5.05	15.87	41.13	0.00	0.00	0.28	0.72	7.24	18.76

Table S32. Chi^2-Test for Athletes Rated by Recruiters vs. Teachers

Variable	$\chi 2$	*df*	*Cramer's V*	*p*
Ego Orientation	10.39	NA	0.30	0.059
Extrinsic Motivation	5.43	NA	0.21	0.381
Fear of Failure	18.45	NA	0.40	< .01**

Variable	*χ2*	*df*	*Cramer's V*	*p*
Hope for Success	6.25	5	0.23	0.282
Intrinsic Motivation	3.15	NA	0.16	0.543
Task Orientation	17.97	NA	0.39	< .01**

Note. df = NA indicates that Monte-Carlo simulation for Chi-squared was applied

Table S33. Observed Frequencies for Athletes Rated by Recruiters vs. Teachers

Rank	H.B	H.T	F.B	F.T	EO.B	EO.T	TO.B	TO.T	IM.B	IM.T	EM.B	EM.T
1	6	7	11	26	9	25	6	2	18	17	1	1
2	6	11	11	16	11	14	1	4	14	22	5	3
3	10	9	8	20	7	14	12	3	9	14	4	1
4	9	25	5	5	13	11	8	9	7	11	5	7
5	10	11	10	1	6	5	10	25	1	5	12	23

Table S34. Expected Frequencies for Athletes Rated by Recruiters vs. Teachers

Rank	H.B	H.T	F.B	F.T	EO.B	EO.T	TO.B	TO.T	IM.B	IM.T	EM.B	EM.T
1	5.40	7.60	15.36	21.64	14.12	19.88	3.32	4.68	14.53	20.47	0.83	1.17
2	7.06	9.94	11.21	15.79	10.38	14.62	2.08	2.92	14.95	21.05	3.32	4.68
3	7.89	11.11	11.63	16.37	8.72	12.28	6.23	8.77	9.55	13.45	2.08	2.92
4	14.12	19.88	4.15	5.85	9.97	14.03	7.06	9.94	7.47	10.53	4.98	7.02
5	8.72	12.28	4.57	6.43	4.57	6.43	14.53	20.47	2.49	3.51	14.53	20.47

Table S35. Chi²-Test for Applicants Rated by Recruiters vs. Teachers

Variable	$\chi2$	*df*	*Cramer's V*	*p*
Ego Orientation	7.08	NA	0.24	0.210
Extrinsic Motivation	11.49	NA	0.31	0.031*
Fear of Failure	10.47	NA	0.30	0.061
Hope for Success	3.08	5	0.16	0.687
Intrinsic Motivation	6.29	NA	0.23	0.267
Task Orientation	2.84	NA	0.16	0.600

Note. df = NA indicates that Monte-Carlo simulation for Chi-squared was applied

Table S36. Observed Frequencies for Applicants Rated by Recruiters vs. Teachers

Rank	H.B	H.T	F.B	F.T	EO.B	EO.T	TO.B	TO.T	IM.B	IM.T	EM.B	EM.T
1	7	7	5	12	2	3	15	22	18	36	1	3
2	6	14	8	8	0	3	21	25	14	19	0	2
3	16	24	21	14	2	0	3	11	6	9	0	5
4	12	18	10	23	3	4	6	9	7	4	9	6
5	6	7	3	9	15	14	3	3	3	1	19	38

Table S37. Expected Frequencies for Applicants Rated by Recruiters vs. Teachers

Rank	H.B	H.T	F.B	F.T	EO.B	EO.T	TO.B	TO.T	IM.B	IM.T	EM.B	EM.T
1	5.69	8.31	6.92	10.08	2.03	2.97	15.05	21.95	21.97	32.03	1.63	2.37

2	8.14	11.86	6.51	9.49	1.22	1.78	18.71	27.29	13.42	19.58	0.81	1.19
3	16.27	23.73	14.24	20.76	0.81	1.19	5.69	8.31	6.10	8.90	2.03	2.97
4	12.20	17.80	13.42	19.58	2.85	4.15	6.10	8.90	4.47	6.53	6.10	8.90
5	5.29	7.71	4.88	7.12	11.80	17.20	2.44	3.56	1.63	2.37	23.19	33.81

RQ 2.2 Differences between Decision Makers – KSV

Table S38. Chi²-Test for Athletes Rated by Scouts vs. Recruiters

Variable	*χ2*	*df*	*Cramer's V*	*p*	*Bonferroni-corrected*
Ego Orientation	18.89	NA	0.49	< .001*	<.0083
Extrinsic Motivation	7.81	NA	0.31	.171	n.s.
Fear of Failure	2.47	NA	0.18	.692	n.s.
Hope for Success	15.10	NA	0.44	< .01*	<.0083
Intrinsic Motivation	18.56	NA	0.48	<.001*	<.0083
Task Orientation	15.77	NA	0.45	< .01*	<.0083

Note. df = NA indicates that Monte-Carlo simulation for Chi-squared was applied

Table S39. Observed Frequencies for Athletes Rated by Recruiters vs. Scouts

Rank	H.B	H.S	F.B	F.S	EO.B	EO.S	TO.B	TO.S	IM.B	IM.S	EM.B	EM.S
1	18	1	0	0	29	5	5	5	17	24	4	0
2	13	11	2	0	8	12	5	13	11	2	9	1

3	12	7	5	1	7	4	13	4	12	3	7	4
4	4	9	9	6	2	7	13	3	8	0	10	10
5	3	1	21	13	4	1	9	3	2	0	15	12

Table S40. Expected Frequencies for Athletes Rated by Recruiters vs. Scouts

Rank	IM.B	IM.S	TO.B	TO.S	EO.B	EO.S	F.B	F.S	EM.B	EM.S	H.B	H.S
1	25.95	15.05	6.33	3.67	21.52	12.48	0.00	0.00	2.53	1.47	12.03	6.97
2	8.23	4.77	11.39	6.61	12.66	7.34	1.27	0.73	6.33	3.67	15.19	8.81
3	9.49	5.51	10.76	6.24	6.96	4.04	3.80	2.20	6.96	4.04	12.03	6.97
4	5.06	2.94	10.13	5.87	5.70	3.30	9.49	5.51	12.66	7.34	8.23	4.77
5	1.27	0.73	7.59	4.41	3.16	1.84	21.52	12.48	17.09	9.91	2.53	1.47

Table S41. Chi^2-Test for Applicants Rated by Scouts vs. Recruiters

Variable	$\chi2$	*df*	*Cramer's V*	*p*
Ego Orientation	5.97	NA	0.28	.301
Extrinsic Motivation	1.37	NA	0.13	.969
Fear of Failure	7.53	NA	0.31	.105
Hope for Success	2.64	NA	0.18	.787
Intrinsic Motivation	4.48	NA	0.24	.375
Task Orientation	3.88	NA	0.22	.444

Note. df = NA indicates that Monte-Carlo simulation for Chi-squared was applied; all Bonferroni-corrections were non significant.

Table S42. Observed Frequencies for Applicants Rated by Recruiters vs. Scouts

Rank	H.B	H.S	F.B	F.S	EO.B	EO.S	TO.B	TO.S	IM.B	IM.S	EM.B	EM.S
1	10	5	0	0	13	12	14	12	34	17	2	1
2	13	6	2	0	10	9	14	9	7	6	1	0
3	16	7	4	2	9	4	13	3	6	3	6	2
4	8	7	13	4	14	3	8	5	3	1	16	9
5	2	3	13	16	3	1	1	0	0	2	20	14

Table S43. Expected Frequencies for Applicants Rated by Recruiters vs. Scouts

Rank	H.B	H.S	F.B	F.S	EO.B	EO.S	TO.B	TO.S	IM.B	IM.S	EM.B	EM.S
1	9.49	5.51	0.00	0.00	15.82	9.18	16.46	9.54	32.28	18.72	1.90	1.10
2	12.03	6.97	1.27	0.73	12.03	6.97	14.56	8.44	8.23	4.77	0.63	0.37
3	14.56	8.44	3.80	2.20	8.23	4.77	10.13	5.87	5.70	3.30	5.06	2.94
4	9.49	5.51	10.76	6.24	10.76	6.24	8.23	4.77	2.53	1.47	15.82	9.18
5	3.16	1.84	18.35	10.65	2.53	1.47	0.63	0.37	1.27	0.73	21.52	12.48

Table S44. Chi^2-Test for Athletes Rated by Scouts vs. Teachers

Variable	*χ2*	*df*	*Cramer's V*	*p*
Ego Orientation	5.87	NA	0.24	0.208
Extrinsic Motivation	5.43	NA	0.23	0.373

Variable	*χ2*	*df*	*Cramer's V*	*p*
Fear of Failure	6.27	NA	0.25	0.281
Hope for Success	14.20	NA	0.37	< .01**
Intrinsic Motivation	5.44	NA	0.23	0.253
Task Orientation	3.51	NA	0.18	0.646

Note. df = NA indicates that Monte-Carlo simulation for Chi-squared was applied

Table S45. Observed Frequencies for Athletes Rated by Scouts vs. Teachers

Rank	H.S	H.T	F.S	F.T	EO.S	EO.T	TO.S	TO.T	IM.S	IM.T	EM.S	EM.T
1	1	18	0	2	5	21	5	19	24	49	0	8
2	11	17	0	3	12	15	13	21	2	14	1	4
3	7	28	1	9	4	18	4	12	3	5	4	5
4	9	7	6	19	7	16	3	14	0	4	10	23
5	1	4	13	19	1	4	3	6	0	2	12	25

Table S46. Expected Frequencies for Athletes Rated by Scouts vs. Teachers

Rank	H.S	H.T	F.S	F.T	EO.S	EO.T	TO.S	TO.T	IM.S	IM.T	EM.S	EM.T
1	5.35	13.65	0.56	1.44	7.32	18.68	6.76	17.24	20.55	52.45	2.25	5.75
2	7.88	20.12	0.84	2.16	7.60	19.40	9.57	24.43	4.50	11.50	1.41	3.59
3	9.85	25.15	2.82	7.18	6.19	15.81	4.50	11.50	2.25	5.75	2.53	6.47
4	4.50	11.50	7.04	17.96	6.48	16.52	4.79	12.21	1.13	2.87	9.29	23.71

5	1.41	3.59	9.01	22.99	1.41	3.59	2.53	6.47	0.56	1.44	10.42	26.58

Table S47. Chi²-Test for Applicants Rated by Scouts vs. Teachers

Variable	*χ2*	*df*	*Cramer's V*	*p*
Ego Orientation	11.49	NA	0.33	0.037*
Extrinsic Motivation	6.99	NA	0.26	0.217
Fear of Failure	8.18	NA	0.28	0.138
Hope for Success	4.23	NA	0.20	0.549
Intrinsic Motivation	5.03	NA	0.22	0.287
Task Orientation	2.16	NA	0.14	0.867

Note. df = NA indicates that Monte-Carlo simulation for Chi-squared was applied

Table S48. Observed Frequencies for Applicants Rated by Scouts vs. Teachers

Rank	H.S	H.T	F.S	F.T	EO.S	EO.T	TO.S	TO.T	IM.S	IM.T	EM.S	EM.T
1	5	15	0	1	12	14	12	29	17	56	1	3
2	6	16	0	2	9	13	9	26	6	5	0	3
3	7	21	2	12	4	22	3	10	3	8	2	17
4	7	19	4	15	3	13	5	7	1	2	9	24
5	3	3	16	20	1	10	0	1	2	3	14	20

Table S49. Expected Frequencies for Applicants Rated by Scouts vs. Teachers

Rank	H.S	H.T	F.S	F.T	EO.S	EO.T	TO.S	TO.T	IM.S	IM.T	EM.S	EM.T
1	5.63	14.37	0.28	0.72	7.32	18.68	11.54	29.46	20.55	52.45	1.13	2.87
2	6.19	15.81	0.56	1.44	6.19	15.81	9.85	25.15	3.10	7.90	0.84	2.16
3	7.88	20.12	3.94	10.06	7.32	18.68	3.66	9.34	3.10	7.90	5.35	13.65
4	7.32	18.68	5.35	13.65	4.50	11.50	3.38	8.62	0.84	2.16	9.29	23.71
5	1.69	4.31	10.14	25.86	3.10	7.90	0.28	0.72	1.41	3.59	9.57	24.43

Table S50. Chi^2-Test for Athletes Rated by Recruiters vs. Teachers

Variable	*χ2*	*df*	*Cramer's V*	*p*
Ego Orientation	15.06	NA	0.35	< .01**
Extrinsic Motivation	8.01	5	0.25	0.156
Fear of Failure	4.87	NA	0.20	0.453
Hope for Success	3.38	NA	0.16	0.510
Intrinsic Motivation	16.05	NA	0.36	< .01**
Task Orientation	15.93	5	0.36	< .01**

Note. df = NA indicates that Monte-Carlo simulation for Chi-squared was applied

Table S51. Observed Frequencies for Athletes Rated by Recruiters vs. Teachers

Rank	H.B	H.T	F.B	F.T	EO.B	EO.T	TO.B	TO.T	IM.B	IM.T	EM.B	EM.T
1	18	18	0	2	29	21	5	19	17	49	4	8
2	13	17	2	3	8	15	5	21	11	14	9	4

3	12	28	5	9	7	18	13	12	12	5	7	5
4	4	7	9	19	2	16	13	14	8	4	10	23
5	3	4	21	19	4	4	9	6	2	2	15	25

Table S52. Expected Frequencies for Athletes Rated by Recruiters vs. Teachers

Rank	H.B	H.T	F.B	F.T	EO.B	EO.T	TO.B	TO.T	IM.B	IM.T	EM.B	EM.T
1	14.52	21.48	0.81	1.19	20.16	29.84	9.68	14.32	26.61	39.39	4.84	7.16
2	12.10	17.90	2.02	2.98	9.27	13.73	10.48	15.52	10.08	14.92	5.24	7.76
3	16.13	23.87	5.65	8.35	10.08	14.92	10.08	14.92	6.85	10.15	4.84	7.16
4	4.44	6.56	11.29	16.71	7.26	10.74	10.89	16.11	4.84	7.16	13.31	19.69
5	2.82	4.18	16.13	23.87	3.23	4.77	6.05	8.95	1.61	2.39	16.13	23.87

Table S53. Chi^2-Test for Applicants Rated by Recruiters vs. Teachers

Variable	$\chi2$	*df*	*Cramer's V*	*p*
Ego Orientation	5.58	5	0.21	0.349
Extrinsic Motivation	3.90	NA	0.18	0.591
Fear of Failure	2.95	NA	0.15	0.750
Hope for Success	3.14	NA	0.16	0.717
Intrinsic Motivation	4.73	NA	0.20	0.332
Task Orientation	5.86	NA	0.22	0.309

Note. df = NA indicates that Monte-Carlo simulation for Chi-squared was applied

Table S54. Observed Frequencies for Applicants Rated by Recruiters vs. Teachers

Rank	H.B	H.T	F.B	F.T	EO.B	EO.T	TO.B	TO.T	IM.B	IM.T	EM.B	EM.T
1	10	15	0	1	13	14	14	29	34	56	2	3
2	13	16	2	2	10	13	14	26	7	5	1	3
3	16	21	4	12	9	22	13	10	6	8	6	17
4	8	19	13	15	14	13	8	7	3	2	16	24
5	2	3	13	20	3	10	1	1	0	3	20	20

Table S55. Expected Frequencies for Applicants Rated by Recruiters vs. Teachers

Rank	H.B	H.T	F.B	F.T	EO.B	EO.T	TO.B	TO.T	IM.B	IM.T	EM.B	EM.T
1	10.08	14.92	0.40	0.60	10.89	16.11	17.34	25.66	36.29	53.71	2.02	2.98
2	11.69	17.31	1.61	2.39	9.27	13.73	16.13	23.87	4.84	7.16	1.61	2.39
3	14.92	22.08	6.45	9.55	12.50	18.50	9.27	13.73	5.65	8.35	9.27	13.73
4	10.89	16.11	11.29	16.71	10.89	16.11	6.05	8.95	2.02	2.98	16.13	23.87
5	2.02	2.98	13.31	19.69	5.24	7.76	0.81	1.19	1.21	1.79	16.13	23.87

RQ 2.3 Differences Between Target Groups – CJ

Table S56. Chi²-Test for Recruiters rating Athletes vs. Applicants

Variable	*χ2*	*df*	*Cramer's V*	*p*
Ego Orientation	4.09	5	0.20	.537

Variable	*χ2*	*df*	*Cramer's V*	*p*
Extrinsic Motivation	3.45	5	0.19	.630
Fear of Failure	11.30	5	0.34	.046*
Hope for Success	6.09	5	0.25	.297
Intrinsic Motivation	7.36	5	0.27	.195
Task Orientation	7.11	5	0.27	.213

Note. df = NA indicates that Monte-Carlo simulation for Chi-squared was applied; all Bonferroni-corrections were non significant.

Table S57. Observed Frequencies for Varying Expertise Recruiters Rating Applicants vs. Athletes

Rank	H.B	H.S	F.B	F.S	EO.B	EO.S	TO.B	TO.S	IM.B	IM.S	EM.B	EM.S
1	13	9	8	10	10	8	6	5	9	11	9	10
2	5	7	12	10	9	15	6	4	11	7	12	6
3	8	7	6	17	9	8	10	9	12	5	6	7
4	5	10	11	4	7	10	10	13	10	13	7	5
5	10	4	8	3	10	5	7	15	5	5	10	13
6	9	13	5	6	5	4	11	4	3	9	6	9

Table S58. Expected Frequencies for Varying Expertise Recruiters Rating Applicants vs. Athletes

Rank	H.B	H.S	F.B	F.S	EO.B	EO.S	TO.B	TO.S	IM.B	IM.S	EM.B	EM.S
1	11.00	11.00	9.00	9.00	9.00	9.00	5.50	5.50	10.00	10.00	9.50	9.50
2	6.00	6.00	11.00	11.00	12.00	12.00	5.00	5.00	9.00	9.00	9.00	9.00

3	7.50	7.50	11.50	11.50	8.50	8.50	9.50	9.50	8.50	8.50	6.50	6.50
4	7.50	7.50	7.50	7.50	8.50	8.50	11.50	11.50	11.50	11.50	6.00	6.00
5	7.00	7.00	5.50	5.50	7.50	7.50	11.00	11.00	5.00	5.00	11.50	11.50
6	11.00	11.00	5.50	5.50	4.50	4.50	7.50	7.50	6.00	6.00	7.50	7.50

Table S59. Chi^2-Test for Scouts rating Athletes vs. Applicants

Variable	*χ2*	*df*	*Cramer's V*	*p*	*Bonferroni-correction*
Ego Orientation	6.99	NA	0.35	.230	n.s.
Extrinsic Motivation	10.90	NA	0.43	.051	n.s.
Fear of Failure	15.59	NA	0.52	< .01*	< .0083*
Hope for Success	8.57	NA	0.38	.131	n.s.
Intrinsic Motivation	10.82	NA	0.43	.054	n.s.
Task Orientation	3.78	NA	0.26	.608	n.s.

Note. df = NA indicates that Monte-Carlo simulation for Chi-squared was applied

Table S60. Observed Frequencies for Varying Expertise Scouts Rating Applicants vs. Athletes

Rank	H.B	H.S	F.B	F.S	EO.B	EO.S	TO.B	TO.S	IM.B	IM.S	EM.B	EM.S
1	7	1	4	16	3	1	8	4	1	6	8	1
2	6	3	9	6	2	8	3	4	4	5	6	4
3	4	4	8	1	6	6	5	4	4	9	4	13
4	3	3	3	2	7	3	6	7	9	5	4	4

5	4	9	3	4	6	8	5	4	6	1	3	4
6	5	9	2	0	5	3	2	6	5	3	4	3

Table S61. Expected Frequencies for Scouts Rating Applicants vs. Athletes

Rank	H.B	H.S	F.B	F.S	EO.B	EO.S	TO.B	TO.S	IM.B	IM.S	EM.B	EM.S
1	4.00	4.00	10.00	10.00	2.00	2.00	6.00	6.00	3.50	3.50	4.50	4.50
2	4.50	4.50	7.50	7.50	5.00	5.00	3.50	3.50	4.50	4.50	5.00	5.00
3	4.00	4.00	4.50	4.50	6.00	6.00	4.50	4.50	6.50	6.50	8.50	8.50
4	3.00	3.00	2.50	2.50	5.00	5.00	6.50	6.50	7.00	7.00	4.00	4.00
5	6.50	6.50	3.50	3.50	7.00	7.00	4.50	4.50	3.50	3.50	3.50	3.50
6	7.00	7.00	1.00	1.00	4.00	4.00	4.00	4.00	4.00	4.00	3.50	3.50

Table S62. Chi^2-Test for Teachers Rating Applicants vs. Athletes

Variable	$\chi 2$	*df*	*Cramer's V*	*p*
Ego Orientation	11.67	5	0.28	0.04*
Extrinsic Motivation	6.25	5	0.21	0.283
Fear of Failure	14.08	5	0.31	0.015*
Hope for Success	11.15	5	0.27	0.048*
Intrinsic Motivation	3.06	5	0.14	0.690
Task Orientation	2.96	5	0.14	0.706

Table S63. Observed Frequencies for Teachers Rating Applicants vs. Athletes

Rank	H.B	H.S	F.B	F.S	EO.B	EO.S	TO.B	TO.S	IM.B	IM.S	EM.B	EM.S
1	16	6	10	26	17	7	11	15	9	14	15	9
2	17	12	16	15	8	7	16	9	13	17	12	15
3	13	17	12	12	17	15	10	11	14	11	17	12
4	12	11	8	7	7	16	14	17	17	12	14	16
5	11	13	15	11	10	19	12	12	15	13	10	8
6	5	15	13	3	15	10	11	10	6	7	6	14

Table S64. Expected Frequencies for Teachers Rating Applicants vs. Athletes

Rank	H.B	H.S	F.B	F.S	EO.B	EO.S	TO.B	TO.S	IM.B	IM.S	EM.B	EM.S
1	11.00	11.00	18.00	18.00	12.00	12.00	13.00	13.00	11.50	11.50	12.00	12.00
2	14.50	14.50	15.50	15.50	7.50	7.50	12.50	12.50	15.00	15.00	13.50	13.50
3	15.00	15.00	12.00	12.00	16.00	16.00	10.50	10.50	12.50	12.50	14.50	14.50
4	11.50	11.50	7.50	7.50	11.50	11.50	15.50	15.50	14.50	14.50	15.00	15.00
5	12.00	12.00	13.00	13.00	14.50	14.50	12.00	12.00	14.00	14.00	9.00	9.00
6	10.00	10.00	8.00	8.00	12.50	12.50	10.50	10.50	6.50	6.50	10.00	10.00

RQ 2.3 Differences Between Target Groups – AHP

Table S65. Chi²-Test for Scouts rating Athletes vs. Applicants

Variable	$\chi 2$	*df*	*Cramer's V*	*p*	*Bonferroni-correction*
Ego Orientation	29.91	NA	0.74	< .001*	< .0083*
Extrinsic Motivation	7.71	NA	0.36	.172	n.s.
Fear of Failure	16.93	NA	0.56	< .01*	< .0083*
Hope for Success	3.02	NA	0.24	.756	n.s.
Intrinsic Motivation	12.09	NA	0.47	.019*	n.s.
Task Orientation	30.38	NA	0.75	< .001*	< .0083*

Note. df = NA indicates that Monte-Carlo simulation for Chi-squared was applied

Table S66. Observed Frequencies for Varying Expertise Scouts Rating Applicants vs. Athletes

Rank	H.B	H.S	F.B	F.S	EO.B	EO.S	TO.B	TO.S	IM.B	IM.S	EM.B	EM.S
1	3	2	1	11	3	4	6	4	15	5	0	1
2	3	4	5	9	1	4	11	1	5	10	1	1
3	8	7	6	3	0	7	7	1	5	6	3	1
4	1	4	3	1	11	2	0	15	2	1	12	5
5	11	8	9	2	1	9	3	4	0	3	1	1
6	1	2	3	1	11	1	0	2	0	2	10	18

Table S67. Expected Frequencies for Varying Expertise Scouts Rating Applicants vs. Athletes

Rank	H.B	H.S	F.B	F.S	EO.B	EO.S	TO.B	TO.S	IM.B	IM.S	EM.B	EM.S
1	2.50	2.50	6.00	6.00	3.50	3.50	5.00	5.00	10.00	10.00	0.50	0.50
2	3.50	3.50	7.00	7.00	2.50	2.50	6.00	6.00	7.50	7.50	1.00	1.00
3	7.50	7.50	4.50	4.50	3.50	3.50	4.00	4.00	5.50	5.50	2.00	2.00
4	2.50	2.50	2.00	2.00	6.50	6.50	7.50	7.50	1.50	1.50	8.50	8.50
5	9.50	9.50	5.50	5.50	5.00	5.00	3.50	3.50	1.50	1.50	1.00	1.00
6	1.50	1.50	2.00	2.00	6.00	6.00	1.00	1.00	1.00	1.00	14.00	14.00

Table S68. Chi^2*-Test for Recruiters rating Athletes vs. Applicants*

Variable	$\chi 2$	*df*	*Cramer's V*	*p*	*Bonferroni-correction*
Ego Orientation	46.58	5	0.69	< .001*	< .0083*
Extrinsic Motivation	11.93	NA	0.35	.023*	n.s.
Fear of Failure	15.78	5	0.40	< .01*	< .0083*
Hope for Success	7.47	5	0.28	.188	n.s.
Intrinsic Motivation	1.59	NA	0.13	.838	n.s.
Task Orientation	43.49	5	0.67	< .001*	< .0083*

Note. df = NA indicates that Monte-Carlo simulation for Chi-squared was applied

Table S69. Observed Frequencies for Varying Expertise Recruiters Rating Applicants vs. Athletes

Rank	H.B	H.S	F.B	F.S	EO.B	EO.S	TO.B	TO.S	IM.B	IM.S	EM.B	EM.S

1	7	6	5	11	2	9	15	6	18	18	1	1
2	6	6	8	11	0	11	21	1	14	14	0	5
3	16	10	21	8	2	7	3	12	6	9	0	4
4	12	9	10	5	3	13	6	8	7	7	9	5
5	6	10	3	10	15	6	3	10	3	1	19	12

Table S70. Expected Frequencies for Recruiters Rating Applicants vs. Athletes

Rank	H.B	H.S	F.B	F.S	EO.B	EO.S	TO.B	TO.S	IM.B	IM.S	EM.B	EM.S
1	6.43	6.57	7.92	8.08	5.44	5.56	10.39	10.61	17.81	18.19	0.99	1.01
2	5.94	6.06	9.40	9.60	5.44	5.56	10.89	11.11	13.86	14.14	2.47	2.53
3	12.87	13.13	14.35	14.65	4.45	4.55	7.42	7.58	7.42	7.58	1.98	2.02
4	10.39	10.61	7.42	7.58	7.92	8.08	6.93	7.07	6.93	7.07	6.93	7.07
5	7.92	8.08	6.43	6.57	10.39	10.61	6.43	6.57	1.98	2.02	15.34	15.66

Table S71. Chi²-Test for Teachers Rating Applicants vs. Athletes

Variable	*χ2*	*df*	*Cramer's V*	*p*
Ego Orientation	91.93	5	0.81	< .001***
Extrinsic Motivation	14.11	NA	0.32	< .01**
Fear of Failure	28.65	NA	0.45	< .001***
Hope for Success	15.20	5	0.33	< .01**
Intrinsic Motivation	15.04	NA	0.33	< .01**
Task Orientation	79.73	5	0.76	< .001***

Note. df = NA indicates that Monte-Carlo simulation for Chi-squared was applied

Table S72. Observed Frequencies for Teachers Rating Applicants vs. Athletes

Rank	H.B	H.S	F.B	F.S	EO.B	EO.S	TO.B	TO.S	IM.B	IM.S	EM.B	EM.S
1	7	7	12	26	3	25	22	2	36	17	3	1
2	14	11	8	16	3	14	25	4	19	22	2	3
3	24	9	14	20	0	14	11	3	9	14	5	1
4	18	25	23	5	4	11	9	9	4	11	6	7
5	7	11	9	1	14	5	3	25	1	5	38	23
6	0	6	4	1	46	0	0	26	1	0	16	34

Table S73. Expected Frequencies for Teachers Rating Applicants vs. Athletes

Rank	H.B	H.S	F.B	F.S	EO.B	EO.S	TO.B	TO.S	IM.B	IM.S	EM.B	EM.S
1	7.05	6.95	19.14	18.86	14.10	13.90	12.09	11.91	26.69	26.31	2.01	1.99
2	12.59	12.41	12.09	11.91	8.56	8.44	14.60	14.40	20.65	20.35	2.52	2.48
3	16.62	16.38	17.12	16.88	7.05	6.95	7.05	6.95	11.58	11.42	3.02	2.98
4	21.65	21.35	14.10	13.90	7.55	7.45	9.06	8.94	7.55	7.45	6.55	6.45
5	9.06	8.94	5.04	4.96	9.57	9.43	14.10	13.90	3.02	2.98	30.72	30.28
6	3.02	2.98	2.52	2.48	23.17	22.83	13.09	12.91	0.50	0.50	25.18	24.82

RQ 2.3 Differences Between Target Groups – CSP

Table S74. Chi²-Test for Scouts rating Athletes vs. Applicants

Variable	$\chi 2$	*df*	*Cramer's V*	*p*
Ego Orientation	4.91	NA	0.29	.304
Extrinsic Motivation	3.07	NA	0.23	.825
Fear of Failure	1.29	NA	0.15	.715
Hope for Success	6.39	NA	0.33	.268
Intrinsic Motivation	6.20	NA	0.33	.152
Task Orientation	8.25	NA	0.38	.118

Note. df = NA indicates that Monte-Carlo simulation for Chi-squared was applied; all Bonferroni-corrections were non significant.

Table S75. Observed Frequencies for Varying Expertise Scouts Rating Applicants vs. Athletes

Rank	H.B	H.S	F.B	F.S	EO.B	EO.S	TO.B	TO.S	IM.B	IM.S	EM.B	EM.S
1	5	1	0	0	12	5	12	5	17	24	1	0
2	6	11	0	0	9	12	9	13	6	2	0	1
3	7	7	2	1	4	4	3	4	3	3	2	4
4	7	9	4	6	3	7	5	3	1	0	9	10
5	3	1	16	13	1	1	0	3	2	0	14	12

Table S76. Expected Frequencies for Varying Expertise Scouts Rating Applicants vs. Athletes

Rank	H.B	H.S	F.B	F.S	EO.B	EO.S	TO.B	TO.S	IM.B	IM.S	EM.B	EM.S
1	3.00	3.00	0.00	0.00	8.50	8.50	8.50	8.50	20.50	20.50	0.50	0.50
2	8.50	8.50	0.00	0.00	10.50	10.50	11.00	11.00	4.00	4.00	0.50	0.50

3	7.00	7.00	1.50	1.50	4.00	4.00	3.50	3.50	3.00	3.00	3.00	3.00
4	8.00	8.00	5.00	5.00	5.00	5.00	4.00	4.00	0.50	0.50	9.50	9.50
5	2.00	2.00	14.50	14.50	1.00	1.00	1.50	1.50	1.00	1.00	13.00	13.00

Table S77. Chi^2-Test for Recruiters rating Athletes vs. Applicants

Variable	$\chi 2$	*df*	*Cramer's V*	*p*	*Bonferroni-correction*
Ego Orientation	16.71	NA	0.41	< .01*	< .0083*
Extrinsic Motivation	9.24	5	0.30	.100	n.s.
Fear of Failure	3.52	NA	0.19	.495	n.s.
Hope for Success	5.39	NA	0.23	.367	n.s.
Intrinsic Motivation	12.83	NA	0.36	< .01*	< .0083*
Task Orientation	21.12	5	0.46	< .001*	< .0083*

Note. df = NA indicates that Monte-Carlo simulation for Chi-squared was applied

Table S78. Observed Frequencies for Varying Expertise Recruiters Rating Applicants vs. Athletes

Rank	H.B	H.S	F.B	F.S	EO.B	EO.S	TO.B	TO.S	IM.B	IM.S	EM.B	EM.S
1	10	18	0	0	13	29	14	5	34	17	2	4
2	13	13	2	2	10	8	14	5	7	11	1	9
3	16	12	4	5	9	7	13	13	6	12	6	7
4	8	4	13	9	14	2	8	13	3	8	16	10
5	2	3	13	21	3	4	1	9	0	2	20	15

Table S79. Expected Frequencies for Recruiters Rating Applicants vs. Athletes

Rank	H.B	H.S	F.B	F.S	EO.B	EO.S	TO.B	TO.S	IM.B	IM.S	EM.B	EM.S
1	14.00	14.00	0.00	0.00	21.00	21.00	9.50	9.50	25.50	25.50	3.00	3.00
2	13.00	13.00	2.00	2.00	9.00	9.00	9.50	9.50	9.00	9.00	5.00	5.00
3	14.00	14.00	4.50	4.50	8.00	8.00	13.00	13.00	9.00	9.00	6.50	6.50
4	6.00	6.00	11.00	11.00	8.00	8.00	10.50	10.50	5.50	5.50	13.00	13.00
5	2.50	2.50	17.00	17.00	3.50	3.50	5.00	5.00	1.00	1.00	17.50	17.50

Table S80. Chi[2]-Test for Teachers Rating Applicants vs. Athletes

Variable	*χ2*	*df*	*Cramer's V*	*p*
Ego Orientation	6.82	5	0.22	0.234
Extrinsic Motivation	9.79	5	0.26	0.081
Fear of Failure	1.54	NA	0.10	0.907
Hope for Success	6.98	NA	0.22	0.137
Intrinsic Motivation	6.29	NA	0.21	0.186
Task Orientation	9.04	NA	0.25	0.101

Note. df = NA indicates that Monte-Carlo simulation for Chi-squared was applied

Table S81. Observed Frequencies for Teachers Rating Applicants vs. Athletes

Rank	H.B	H.S	F.B	F.S	EO.B	EO.S	TO.B	TO.S	IM.B	IM.S	EM.B	EM.S

1	15	18	1	2	14	21	29	19	56	49	3	8
2	16	17	2	3	13	15	26	21	5	14	3	4
3	21	28	12	9	22	18	10	12	8	5	17	5
4	19	7	15	19	13	16	7	14	2	4	24	23
5	3	4	20	19	10	4	1	6	3	2	20	25

Table S82. Expected Frequencies for Teachers Rating Applicants vs. Athletes

Rank	H.B	H.S	F.B	F.S	EO.B	EO.S	TO.B	TO.S	IM.B	IM.S	EM.B	EM.S
1	16.50	16.50	1.50	1.50	17.50	17.50	24.00	24.00	52.50	52.50	5.50	5.50
2	16.50	16.50	2.50	2.50	14.00	14.00	23.50	23.50	9.50	9.50	3.50	3.50
3	24.50	24.50	10.50	10.50	20.00	20.00	11.00	11.00	6.50	6.50	11.00	11.00
4	13.00	13.00	17.00	17.00	14.50	14.50	10.50	10.50	3.00	3.00	23.50	23.50
5	3.50	3.50	19.50	19.50	7.00	7.00	3.50	3.50	2.50	2.50	22.50	22.50

RQ 2.4 Differences between Assessment Methods

Table S83. Hope for Success in Athletes

Rank	conjoint	ahp	csp
1	13<br>21.24	15<br>20.51	35<br>21.24
2	21<br>27.31	19<br>26.37	41<br>27.31
3	27<br>33.05	27<br>31.91	44<br>33.05
4	24<br>27.31	42<br>26.37	15<br>27.31
5	26<br>18.88	21<br>18.23	9<br>18.88
6	34<br>17.20	16<br>16.60	1<br>17.20

Note. Upper Number: Observed Frequencies, Lower Number: Expected Frequencies after Monte Carlo simulation.

Table S84. Hope for Success in Applicants

Rank	conjoint	ahp	csp
1	34<br>26.33	17<br>26.33	28<br>26.33
2	28<br>28.33	23<br>28.33	34<br>28.33
3	24<br>37.33	48<br>37.33	40<br>37.33
4	17<br>29.67	41<br>29.67	31<br>29.67
5	25<br>16.00	14<br>16.00	9<br>16.00
6	17<br>7.33	2<br>7.33	3<br>7.33

Note. Upper Number: Observed Frequencies, Lower Number: Expected Frequencies after Monte Carlo simulation.

Table S85. Fear of Failure in Athletes

Rank	conjoint	ahp	csp
1	48<br>31.36	43<br>30.28	2<br>31.36
2	31<br>23.94	36<br>23.12	4<br>23.94
3	27<br>24.28	31<br>23.44	14<br>24.28
4	12<br>15.85	12<br>15.30	23<br>15.85
5	18<br>23.27	12<br>22.47	39<br>23.27
6	9<br>26.30	6<br>25.40	63<br>26.30

Note. Upper Number: Observed Frequencies, Lower Number: Expected Frequencies after Monte Carlo simulation.

Table S86. Fear of Failure in Applicants

Rank	conjoint	ahp	csp
1	21<br>13.33	18<br>13.33	1<br>13.33
2	34<br>19.67	21<br>19.67	4<br>19.67
3	24<br>26.67	41<br>26.67	15<br>26.67
4	22<br>30.00	42<br>30.00	26<br>30.00
5	25<br>26.00	15<br>26.00	38<br>26.00
6	19<br>29.33	8<br>29.33	61<br>29.33

Note. Upper Number: Observed Frequencies, Lower Number: Expected Frequencies after Monte Carlo simulation.

Table S87. Ego Orientation in Athletes

Rank	conjoint	ahp	csp
1	16<br>36.42	38<br>35.16	54<br>36.42
2	29<br>29.67	27<br>28.65	32<br>29.67
3	28<br>27.99	27<br>27.02	28<br>27.99
4	25<br>25.29	31<br>24.42	19<br>25.29
5	30<br>17.87	13<br>17.26	10<br>17.87
6	17<br>7.76	4<br>7.49	2<br>7.76

Note. Upper Number: Observed Frequencies, Lower Number: Expected Frequencies after Monte Carlo simulation.

Table S88. Ego Orientation in Applicants

Rank	conjoint	ahp	csp
1	28<br>23.33	8<br>23.33	34<br>23.33
2	18<br>17.33	4<br>17.33	30<br>17.33
3	31<br>22.33	2<br>22.33	34<br>22.33
4	20<br>19.00	8<br>19.00	29<br>19.00
5	24<br>25.67	40<br>25.67	13<br>25.67
6	24<br>37.33	83<br>37.33	5<br>37.33

Note. Upper Number: Observed Frequencies, Lower Number: Expected Frequencies after Monte Carlo simulation.

Table S89. Task Orientation in Athletes

Rank	conjoint	ahp	csp
1	24<br>21.24	12<br>20.51	27<br>21.24
2	15<br>19.22	7<br>18.56	35<br>19.22
3	23<br>21.58	13<br>20.84	28<br>21.58
4	36<br>27.99	20<br>27.02	27<br>27.99
5	28<br>32.37	49<br>31.26	19<br>32.37
6	19<br>22.59	39<br>21.81	9<br>22.59

Note. Upper Number: Observed Frequencies, Lower Number: Expected Frequencies after Monte Carlo simulation.

Table S90. Task Orientation in Applicants

Rank	conjoint	ahp	csp
1	23<br>39.33	43<br>39.33	52<br>39.33
2	24<br>42.67	57<br>42.67	47<br>42.67
3	24<br>23.00	21<br>23.00	24<br>23.00
4	30<br>21.33	18<br>21.33	16<br>21.33
5	21<br>10.67	6<br>10.67	5<br>10.67
6	23<br>8.00	0<br>8.00	1<br>8.00

Note. Upper Number: Observed Frequencies, Lower Number: Expected Frequencies after Monte Carlo simulation.

Table S91. Intrinsic Motivation in Athletes

Rank	conjoint	ahp	csp
1	30<br>52.27	40<br>50.47	85<br>52.27
2	26<br>32.71	44<br>31.58	27<br>32.71
3	24<br>23.94	28<br>23.12	19<br>23.94
4	30<br>20.57	20<br>19.86	11<br>20.57
5	18<br>9.10	6<br>8.79	3<br>9.10
6	17<br>6.41	2<br>6.19	0<br>6.41

Note. Upper Number: Observed Frequencies, Lower Number: Expected Frequencies after Monte Carlo simulation.

Table S92. Intrinsic Motivation in Applicants

Rank	conjoint	ahp	csp
1	18<br>63.67	69<br>63.67	104<br>63.67
2	26<br>27.00	38<br>27.00	17<br>27.00
3	29<br>21.33	20<br>21.33	15<br>21.33
4	35<br>16.67	11<br>16.67	4<br>16.67
5	24<br>11.67	6<br>11.67	5<br>11.67
6	13<br>4.67	1<br>4.67	0<br>4.67

Note. Upper Number: Observed Frequencies, Lower Number: Expected Frequencies after Monte Carlo simulation.

Table S93. Extrinsic Motivation in Athletes

Rank	conjoint	ahp	csp
1	20<br>11.80	3<br>11.40	12<br>11.80
2	24<br>15.17	9<br>14.65	12<br>15.17
3	30<br>17.20	6<br>16.60	15<br>17.20
4	24<br>24.95	12<br>24.09	38<br>24.95
5	23<br>39.12	40<br>37.77	53<br>39.12
6	24<br>36.76	70<br>35.49	15<br>36.76

Note. Upper Number: Observed Frequencies, Lower Number: Expected Frequencies after Monte Carlo simulation.

Table S94. Extrinsic Motivation in Applicants

Rank	conjoint	ahp	csp
1	32<br>14.00	4<br>14.00	6<br>14.00
2	28<br>11.67	3<br>11.67	4<br>11.67
3	24<br>17.33	8<br>17.33	20<br>17.33
4	23<br>28.67	16<br>28.67	47<br>28.67
5	22<br>48.00	69<br>48.00	53<br>48.00
6	16<br>25.33	45<br>25.33	15<br>25.33

Note. Upper Number: Observed Frequencies, Lower Number: Expected Frequencies after Monte Carlo simulation.
